# Formosulfathiazole: A Structural Revision

**DOI:** 10.1002/cplu.202500406

**Published:** 2025-09-03

**Authors:** Claudio Maestri, Toni Grell, Fabio Travagin, Christian R. Göb, Michele Castaldi, Ivana Miletto, Geo Paul, Silvia Zampini, Marco Vandone, Valentina Colombo, Giovanni B. Giovenzana

**Affiliations:** ^1^ Dipartimento di Scienze del Farmaco Università del Piemonte Orientale, Largo Guido Donegani 2 Novara (NO) 28100 Italy; ^2^ PRC Ticinum Lab S.r.l. Via Bovio 6 Novara (NO) 28100 Italy; ^3^ Dipartimento di Chimica Università degli Studi di Milano & INSTM UdR Milano Via Golgi 19 Milano 20133 Italy; ^4^ Rigaku Europe SE Hugenottenallee 167 63263 Neu‐Isenburg Germany; ^5^ Chemelectiva S.r.l. Strada Privata Due Ponti 12 Novara (NO) 28100 Italy; ^6^ Dipartimento di Scienze e Innovazione Tecnologica Università del Piemonte Orientale Viale Teresa Michel 11 Alessandria (AL) 15121 Italy

**Keywords:** cyclodimer, cyclophane, electron diffraction, formosulfathiazole, structural revision

## Abstract

Formosulfathiazole (FSTz) is a synthetic active pharmaceutical ingredient (API) prepared by condensation of sulfathiazole with formaldehyde. Originally described for the first time in 1948, it is currently used for the treatment of bacterial and protozoal infections in cattle and pets, acting as a prodrug slowly releasing the sulfamidic sulfathiazole and formaldehyde. A systematic analysis of FSTz allowed to revise the originally believed undefined polymeric structure and uncovered the intriguing cyclophane skeleton of a well‐defined cyclodimeric condensation product.

## Graphical abstract

Formosulfathiazole (FSTz), a synthetic drug developed in 1948, is used to treat bacterial and protozoal infections in animals by slowly releasing sulfathiazole and formaldehyde. Structural analysis revealed that, contrary to earlier beliefs, FSTz features a well‐defined cyclophane cyclodimeric structure rather than an undefined polymer.

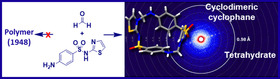

## 1 Introduction

Formosulfathiazole (FSTz), previously known as formocibazol or intraformazol, is a veterinary antibacterial active pharmaceutical ingredient (API) belonging to the sulfamidic family.^[^ ^1^ ^,^ ^2^ ^]^ FSTz is prepared by condensation of sulfathiazole with formaldehyde. As other sulfamidics, FSTz exerts its bacteriostatic action by inhibiting the transformation of *p*‐aminobenzoic acid into dihydrofolic acid.^[^ ^3^ ^]^ Moreover, after administration, it releases formaldehyde in the organism, then acting as a powerful topical disinfectant/microbicidal.^[^ ^4^ ^,^ ^5^ ^]^

FSTz can be administered both locally or orally to cattle and domestic animals to eradicate bacteria (gram positive,^[^ ^6^, ^7^ ^–^ ^9^ ^]^ negative,^[^ ^6^ ^,^ ^10^ ^]^ and intracellular)^[^ ^4^ ^]^ and protozoa^[^ ^11^ ^]^ (including coccidia and toxoplasma). FSTz may be used in combination with other antibiotic APIs.^[^ ^12^ ^]^

FSTz is also used topically for the treatment of skin and soft tissue lesions and for the intrauterine treatment of postpartum endometritis in different animals. Its poor absorption allows the slow delivery of a high local dose of FSTz, optimal for local antibacterial treatments.^[^ ^8^ ^,^ ^13^ ^]^

FSTz is currently described in the literature as an undefined polymer of sulfathiazole and formaldehyde, registered as CAS number 12041‐72‐4. Interestingly, and rather surprisingly, despite 77 years having passed since its initial synthesis, no significant efforts have been made to elucidate its structure, as evidenced by the complete lack of structural details in the literature.^[^ ^14^ ^]^

## 2 Results and Discussion

FSTz was prepared following the procedure reported by Hartmann et al.^[^ ^6^ ^]^ (Scheme S1, Supporting Information). FSTz is a white solid, reportedly melting at 265‐270 °C with decomposition.^[^ ^15^ ^]^ Its high reticular stability in the solid state is evidenced by its insolubility in most solvents and mixtures thereof, with the sole exception of dimethylsulfoxide (DMSO). Stock solutions of FSTz in DMSO can be prepared at room temperature, as even moderate heating triggers the decomposition of FSTz. Notably, partial decomposition is observed even after a few hours of storage at room temperature. For analytical purposes, freshly prepared solutions of FSTz in DMSO at room temperature were used in this work.

RP‐HPLC analysis of the DMSO solution of FSTz, routinely used to assess its identity and purity, displays a single major peak (Figure S1, Supporting Information, average area% ≈93%), along with trace amounts of related species (among them sulfathiazole), likely arising from the ensuing decomposition. The presence of a single major chemical species, as suggested by chromatographic analysis, is hardly compatible with an undefined polymeric structure, typically exhibiting a substantial degree of polydispersity. The NMR analysis of a freshly prepared solution of FSTz in DMSO‐d_6_ further supports this observation, showing a single set of signals in both ^1^H and ^13^C spectra (Figures S2–S8 and Table S1, Supporting Information), indicative of a highly symmetrical structure. The characteristic signals of sulfathiazole are accompanied by an additional signal of a methylene group, clearly derived from the condensation with formaldehyde. The ^1^H‐NMR integrals of the signals of the methylene group and of the sulfathiazole moiety give a net 1:1 ratio, suggesting a perfect equimolar condensation process of the two starting materials. The sharp signals in the NMR spectra are not compatible with the polymeric structure reported by CAS.

Electrospray Iionization high‐resolution mass spectrometry (HRMS) analysis (Figure S9, Supporting Information) shows a quite complex spectrum, containing a major peak of the protonated molecular ion corresponding to the formula ([C_20_H_18_N_6_O_4_S_4_ + H^+^]). This ion may correspond to a [2 + 2] condensation product, that is a dimer of sulfathiazole and formaldehyde, with the elimination of two water molecules. The presence of further multiple fragmentation peaks may be explained by a rapid degradation of the thermally unstable FSTz in the high temperature environment (350 °C) of the mass spectrometer ion source.

**Figure 1:**
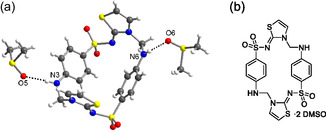
a) Crystal structure (asymmetric unit only) of the DMSO solvate (FSTz·2DMSO) showing the symmetrically independent formosulfathiazole dimer and two independent DMSO molecules. Color code: C, gray; N, blue; O, red; S, yellow; H, white. Selected H‐bonds distances: N3(‐H)···O5 = 2.846(2) Å, N6(‐H)···O6 = 2.897(2) Å. b) Chemical structure of FSTz·2DMSO.

Prolonged standing of a saturated solution of FSTz in DMSO at room temperature results in the formation of small colorless crystals, suitable for single‐crystal X‐ray diffraction (SC‐XRD). The analysis reveals a solvatomorph of FSTz with one molecule of the API which cocrystallized with two DMSO molecules (FSTz·2DMSO) in general position (space group *P*2_1_/*n*) linked by hydrogen bonds (**Table** **1**). The molecule is constituted by a cyclodimeric structure, which is based on an unusual cyclophane skeleton (**Figures** **1**, S10, S11, Table S2, Supporting Information). The sulfathiazole subunits are linked in a head‐to‐tail fashion via methylene groups, bridging the amine group on the benzene ring with the endocyclic nitrogen atom of the thiazole ring in its 2‐imino‐(3*H*)‐thiazole form, leading to an approximate *C*_2_ symmetry of the dimer. Notably, the thiazole rings adopt the nonaromatic 2‐iminothiazoline tautomeric form. Although the loss of aromaticity in the thiazole ring may appear unexpected, sulfathiazole itself is known to adopt this tautomeric form in several crystalline modifications available in the Cambridge structural database (see Table S3, Supporting Information).^[^ ^16^ ^]^ This suggests that solid state interactions play a key role in its stabilization.

**Table 1:** Crystallographic data for FSTz·2DMSO and FSTz.4H_2_O.

Compound	FSTz·2DMSO	FSTz.4H_2_O
CCDC	2444187	2430519
Formula	C_24_H_30_N_6_O_6_S_6_	C_20_H_26_N_6_O_8_S_4_
FW	690.90	606.73
Crystal system	monoclinic	monoclinic
Space group	*P*2_1_/n	*C*2/*c*
a (Å)	11.2812(2)	27.799(16)
b (Å)	23.4425(3)	10.981(2)
c (Å)	11.9842(2)	17.503(3)
*α* (°)	90	90
*β* (°)	107.059(2)	96.88(2)
*γ* (°)	90	90
V (Å^3^)	3029.89(9)	5305(3)
Z	4	8
Radiation	CuK*α* (*λ* =1.54184)	electron (*λ* = 0.0251)
Temperature (K)	299(2)	100
*ρ*_(calcd)_ (g·cm^−3^)	1.515	1.519
μ (mm^−1^)	4.602	
F(000)	1440.0	909.0
Crystal size (mm^3^)	0.12 × 0.025 × 0.02	0.0010 × 0.0006 × 0.0003
*2Θ* range (°)	7.542 to 152.51	0.104 to 1.798
Limiting indices	−14 ≤ *h* ≤ 13,−28 ≤ *k* ≤ 29,−14 ≤ *l* ≤ 14	−34 ≤ *h* ≤ 34,−13 ≤ *k* ≤ 13,−21 ≤ *l* ≤ 21
Reflns collected/uniquea)	67,651/6139 [R_int_ = 0.0503]	39,299/5421 [R_int_ = 0.2888, R_sigma_ = 0.1402]
Data/restraints/param	6139/2/391	5421/282/350
Completeness (%) [to *θ*]	100.0 (67.684°)	
Max. and min. transmission	1.00000 and 0.43285	
Final R indices [I > 2*σ*(I)]b)	R_1_ = 0.0344, wR_2_ = 0.1009	R_1_ = 0.1868, wR_2_ = 0.4331
R indices (all data)	R_1_ = 0.0379, wR_2_ = 0.1036	R_1_ = 0.2157, wR_2_ = 0.4529
GooF on F2c)	1.061	1.208
Largest diff peak and hole (e·Å^−3^)	0.34 and −0.37	0.24/−0.24

a) R_int_ = *Σ*|F_o_^2^ − F_o_^2^(mean)|/*Σ*F_o_^2^.

b) R_1_ = *Σ*||F_o_| − |F_c_||/*Σ*|F_o_|, wR_2_ = {*Σ*[*w*(F_o_^2^ − F_c_^2^)^2^]/*Σ*[*w*(F_o_^2^)^2^]}^1/2^.

c) GooF = {S/(*n −p*)}^1/2^ = {*Σ*[*w*(F_o_^2^ − F_c_^2^)^2^]/(*n − p*)}^1/2^.

The NMR data obtained in DMSO‐d_6_ perfectly fit with the symmetry reported in Figure 1. Both ^1^H and ^13^C NMR spectra (Figures S2–S8, Supporting Information) show a single set of signals, consistent with the *C*_2_‐symmetry of the molecule. The UV spectrum of the solution (Figure S12A, Supporting Information) shows absorption bands at 263 nm and 288 nm, attributed to n → π* and *π* → π* transitions. A broad fluorescence band centered at 337 nm (shoulder at 430 nm) is obtained upon excitation of FSTz at 288 nm (Figure S12B, Supporting Information). No clear evidence of *π*‐stacking of the aromatic rings may be appreciated, in accordance with their nonparallel arrangement found in the crystal structure.

The cyclodimeric structure of FSTz shown in Figure 1 is fully compatible with the analytical data determined on solution of FSTz in DMSO or from the solid crystallized from it. However, it is mandatory to ascertain whether this structural motif is an intrinsic feature of solid FSTz or an artifact arising from a solution‐phase equilibrium involving oligo/polymeric species.

**Figure 2:**
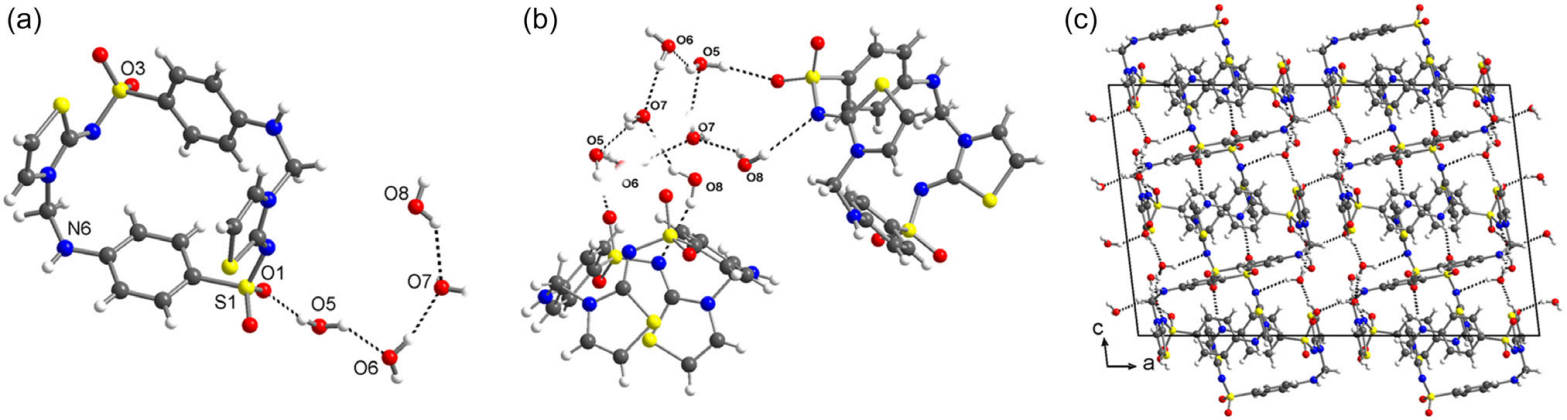
a) Asymmetric unit of the crystallographic structure of formosulfathiazole tetrahydrate (FSTz·4H_2_O) showing the conformation of FSTz dimer and the four water molecules. b) Enlargement of a portion of the crystal packing showing the water molecules forming an H‐bonded octameric cluster (see SI for further details). c) Crystal packing viewed down the b axis highlighting the intricate pattern of water‐mediated hydrogen bonds. Color code: C, gray; N, blue; O, red; S, yellow; H, white. H‐bonds.

It is therefore essential to obtain structural data on the bulk material prior to solubilization. An initial analysis with powder X‐ray diffraction (PXRD) shows that solid pristine FSTz is crystalline and, as expected, its PXRD profile does not correspond to the one already observed for the DMSO solvate (Figure S13, Supporting Information). Notably, indexing^[^ ^17^ ^]^ (Table S4, Figure S14, Supporting Information) and Le Bail refinement^[^ ^18^ ^]^ of the PXRD pattern confirmed the bulk sample's purity, the lattice parameters being refined to a monoclinic *C* unit cell with volume ≈10% larger than what is expected for a crystal structure with a single FSTz dimer in a general position (Z = 8, Z′ = 1). Since ab initio structure solution from PXRD data proved unsuccessful, we employed electron diffraction (ED) ‐ now widely used for structural characterization ‐ to analyze the small crystals in the bulk FSTz sample. The experiment revealed that pristine formosulfathiazole also contains the same cyclophane cyclodimer and is in fact a tetrahydrate with the formula FSTz·4H_2_O (**Figure** **2**, Table 1, Tables S4–S6, Figures S15–S19, Supporting Information), which crystallizes in space group *C*2/*c* with all molecules in the general position. The molecular structure of the cyclodimer has an almost identical conformation compared to the molecule in the DMSO solvatomorph (Figure S20, Supporting Information).

In between the FSTz molecules, cocrystallized water molecules form a hydrogen‐bonded octamer with a bicyclo [2.2.2]octane‐like structure (Figure S16, Supporting Information, H‐bond lengths 2.73(2)−2.92(2) Å). These clusters furthermore connect four different FSTz molecules via hydrogen bonds ranging from 2.86(1) to 3.29(2) Å (Figure S17, Supporting Information), leading to a stacking along the crystallographic [010] direction (Figure 2b). Notably, two cyclodimeric rings are oriented face‐to‐face through an inversion center, further stabilizing the network via weak intermolecular interactions between the thiazole rings (Figures S18a, b, Supporting Information). Additionally, intermolecular hydrogen bonding is observed between adjacent FSTz molecules, involving the NH and the SO_2_ group of neighboring molecules (N6(−H)···O3 = 2.984 Å). Interestingly, the void space after removing all water molecules from the structure is ≈20% that of the unit cell, with channels that widen and narrow along the crystallographic [001] direction (Figure S19, Supporting Information). The presence of these distinct water channels may also be the reason why this compound gave no rise to diffraction when investigated with ED at room temperature, as the high vacuum conditions of the electron diffractometer cause the structure to collapse. When ED measurements were conducted at 100 K, the vapor pressure was low enough so that all cocrystallized water molecules and crystallinity of the sample are maintained and diffraction was observed.

ED is not fully representative of the bulk sample, as it gives information of only a tiny fraction. Therefore, a Rietveld refinement was performed on the PXRD pattern of pristine FSTz using the structural model obtained from ED. The successful refinement (Figure S21, Supporting Information) confirms that the bulk sample corresponds to the ED‐structure and that the pristine API is indeed the tetrahydrate FSTz·4H_2_O.

The crystallographic analysis indeed confirms that the cyclodimeric structure of FSTz is already present in the solid state and remains unchanged upon dissolution in DMSO at room temperature, provided that higher temperatures are avoided when the API is solubilized. Additionally, these analyses confirmed that pristine formosulfathiazole exists as a hydrate form. The degree of hydration of FSTz·4H_2_O, was validated by Karl‐Fischer titration (experimental 11.42 ± 0.16% vs*.* calculated 11.89%, Table S7, Supporting Information) and TGA analysis, showing a weight loss between 30 and 100 °C (experimental 10.37% vs*.* calculated 11.89%, Figure S22, Supporting Information). The derivative signal of the thermogravimetry reveals that dehydration occurs in two steps; however, these are closely overlapping and cannot be fully resolved, even at a slower heating rate (Figure S22, Supporting Information). To gain deeper insight into the dehydration process, we conducted variable‐temperature PXRD (VT‐PXRD) experiments. The results (Figures S23–S27, Supporting Information) show that FSTz·4H_2_O retains good crystallinity and remains stable between room temperature and ≈100 °C (Figure S23, Supporting Information), consistent with the thermal behavior observed in the TGA data. No intermediate phases with distinct hydration levels were detected, indicating that dehydration does not proceed in multiple discrete steps. Once dehydration is complete, a new phase appears, characterized by very low crystallinity, which prevents the extraction of structural information by PXRD methods (Figure S24, Supporting Information). This poorly crystalline phase persists up to ≈230 °C, beyond which the sample becomes brown and amorphous (Figure S25, Supporting Information). Importantly, the original tetrahydrate phase cannot be recovered by simply returning the sample—either previously heated to 100 °C or dehydrated under vacuum at 40 °C overnight—to room temperature and exposing it to air, even over extended periods (Figure S26, Supporting Information). However, crystallinity is restored when the sample is immersed in or wetted with water, regenerating the FSTz·4H_2_O crystal form (Figure S27, Supporting Information). This indicates that dehydration does not irreversibly disrupt the FSTz cyclodimer, unless higher temperatures are reached. Degradation is indeed observed at T_dec_ = 270 °C (S22, T_peak_), a value consistent with the early literature reporting that FSTz melts at 265–270 °C with decomposition.^[^ ^15^ ^]^ These observations suggest that the low‐crystalline intermediate phase likely corresponds to the anhydrous form of FSTz and that the tetrahydrate exhibits notable solid‐state stability, especially when compared to its instability in solution.

Fourier transform infra red spectroscopy allows to discern the octamer of hydrogen‐bonded water molecules (Figure S28, Supporting Information), showing a broad signal at 3507 cm^−1^ (*ν*_OH_) and at 1641 cm^−1^ (δ_HOH_), along with further signals which are compatible with the proposed structure (see SI). It is worth noting that in the high frequency region typical of N—H stretching modes, only the signal attributed to the N—H stretching vibration of the amine group on the benzene ring was observed (3353 cm^−1^), whilst the signal of a secondary sulfonamide^[^ ^19^ ^,^ ^20^ ^]^ (expected at ca. 3255–3265 cm^−1^) is not present, thus confirming the results from diffraction analyses of the thiazole ring in the nonaromatic 2‐iminothiazoline tautomeric form.

Lastly, ^13^C CPMAS NMR spectrum (Figure S30, Supporting Information) shows two sets of peaks for each carbon atom in FSTz·4H_2_O, which is compatible with the chemical inequivalence of the cyclodimeric halves in the asymmetric unit. Since ^13^C chemical shifts are very sensitive to molecular geometry, conformation, and the local molecular environment, nonequivalent carbon sites in the asymmetric unit within the crystallographic unit cell gave rise to ‘crystallographic splitting’ of the ^13^C resonances. The ^13^C chemical shifts and assignments are given in Table S8, Supporting Information. Each carbon atoms of the cyclodimer resonate at a single frequency, however, the differences, in ppm, between the similar carbon pairs are rather small. The crystallographic splitting for carbons 4 and 4^′^ is not visible in the ^13^C CPMAS NMR spectrum. Further evidence for the existence of water in FSTz·4H_2_O comes from the ^1^H MAS NMR data (Figure S31, Supporting Information), which clearly distinguished the presence of different types of protons and moreover, showed that the relative proportion of the population distribution of ^1^H sites, obtained from the quantitative ^1^H MAS NMR data analysis sites, matches the expected values (Table S9, Supporting Information).

**Scheme 1:**
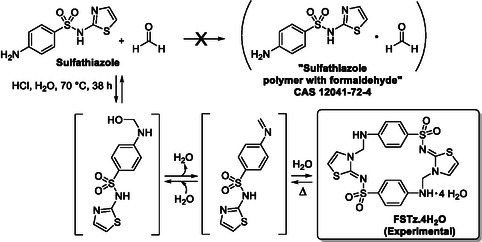
Formosulfathiazole (FSTz): synthesis, originally proposed and revised experimental structure.

The analytical data collected for FSTz, either in the (native) solid state or in solution of DMSO allows to revise the originally alleged polymeric structure and to draw a clearer scenario, summarized in **Scheme** **1**.

The reaction of sulfathiazole with formaldehyde likely starts with the initial formation of an hemiaminal, eventually evolving to an unstable intermediate imine. The latter undergoes an intermolecular head‐to‐tail dimerization yielding FSTz, which precipitates as the tetrahydrate in microcrystalline form. The aminal groups formed during the dimerization step are known to be prone to thermal or hydrolytic dissociation phenomena.^[^ ^21^ ^]^ Similarly, the other steps of the reaction sequence are well‐known to be reversible, making the overall reverse reaction feasible. Indeed, this is demonstrated by the observed decomposition of the API by moderate heating or even prolonged standing of a solution at room temperature. This is also in accordance with the mode of action of FSTz, which, when ingested, decomposes at body temperatures to liberate its parent compounds, exerting the antibacterial effects.

## 3 Conclusion

The structural determination of formosulfathiazole (FSTz), the API of a veterinary antibacterial, has been carried out with a systematic and comprehensive combination of analytical techniques. This work, summarized in Scheme 1, allowed to revise the originally believed undefined polymeric structure after 77 years from the first literature report and to assign to FSTz the structure of a cyclodimeric condensation product of the starting materials (sulfathiazole and formaldehyde) in the solid form of a tetrahydrate. FSTz embodies an intriguing cyclophane skeleton, quite unusual in APIs, and its proven instability in solution is substantial for its pharmacological activity as a slow‐release antibacterial.

## 4 Experimental Section

Experimental details for the preparation (synthetic procedure) of FSTz and its full characterization (HPLC trace, ^1^H‐ and ^13^C‐NMR spectra, HRMS spectrum, SC‐XRD, UV–Vis spectra, PXRD, ED analysis, Karl‐Fischer analysis, thermogravimetric analysis, VT‐PXRD, ATR‐IR spectra, and ssNMR spectra) are provided in the Supporting Information. CCDC 2430519 (FSTz·4H_2_O) and CCDC 2444187(FSTz·2DMSO) contain the supplementary crystallographic data for this article. These data and additional information can be obtained free of charge via https://summary.ccdc.cam.ac.uk/structure‐summary‐form (or from the Cambridge Crystallographic Data Centre, 12 Union Road, Cambridge CB2 1EZ, UK; fax: (+44)1223‐336−033; or deposit@ccdc.cam.uk). Data availability: the original data are available from the authors upon request. The authors have cited additional references within the Supporting Information (SI).^[^ ^18^ ^,^ ^22^, ^23^, ^24^, ^25^, ^26^, ^27^, ^28^, ^29^, ^30^, ^31^ ^–^ ^32^ ^]^

## Conflict of Interest

The authors declare no conflict of interest.

## Supporting information

Supplementary Material

## Data Availability

The data that supports the findings of this study are available in the supplementary material of this article and are available from the authors upon request.
