# Supplementary material for: Formosulfathiazole: A Structural Revision
Source: Chempluschem. 2025 Sep 3;90(11):e202500406. doi: 10.1002/cplu.202500406 (PMC12605753; doi:10.1002/cplu.202500406)
Supplement: Supplementary file 1 — Supplementary Material [file CPLU-90-e202500406-s001.pdf]

Supporting Information
©Wiley-VCH 2021
69451 Weinheim, Germany

Formosulfathiazole: a Structural Revision

Claudio Maestri,[a,b] Toni Grell,[c] Fabio Travagin,[a] Christian R. Göb,[d] Michele Castaldi,[e] Ivana Miletto,[a] Geo Paul,[f] Silvia Zampini,[c] Marco Vandone,[c] Valentina Colombo,[c]* Giovanni B. Giovenzana[a]*

[a] Mr. C. Maestri, Dr. F. Travagin, Prof. I. Miletto, Prof. G. B. Giovenzana
Dipartimento di Scienze del Farmaco
Università del Piemonte Orientale
Largo Guido Donegani 2, 28100 Novara (NO), Italy
E-mail: giovannibattista.giovenzana@uniupo.it

[b] Mr. C. Maestri
PRC Ticinum Lab S.r.l.
Via Bovio 6, 28100 Novara (NO), Italy

[c] Dr. T. Grell, Ms. S. Zampini, Mr. M. Vandone, Prof. V. Colombo
Dipartimento di Chimica
Università degli Studi di Milano & INSTM UdR Milano
Via Golgi 19, 20133 Milano, Italy

[d] Dr. C. R. Göb
Rigaku Europe SE
Hugenottenallee 167, 63263 Neu-Isenburg, Germany

[e] Dr. M. Castaldi
Chemelectiva S.r.l.
Strada Privata Due Ponti 12, 28100 Novara (NO), Italy

[f] Dr. G. Paul
Dipartimento di Scienze e Innovazione Tecnologica
Università del Piemonte Orientale
Viale Teresa Michel 11, 15121 Alessandria (AL), Italy

Abstract: Formosulfathiazole (FSTz) is a synthetic active pharmaceutical ingredient (API) prepared by condensation of sulfathiazole with formaldehyde. Originally described for the first time in 1948, it is currently used for the treatment of bacterial and protozoal infections in cattle and pets, acting as a pro-drug slowly releasing the sulfamidic sulfathiazole and formaldehyde. A systematic analysis of FSTz allowed to revise the originally believed undefined polymeric structure and uncovered the intriguing cyclophane skeleton of a well-defined cyclodimeric condensation product.

DOI: 10.1002/anie.2021XXXXX

Table of Contents

Table of Contents ... 2
Experimental Procedures ... 2
Preparation of FSTz ... 2
HPLC chromatogram ... 3
Full assignment of ls-NMR ^{1}H and ^{13}C signals of FSTz ... 4
HRMS analysis ... 8
Single crystal X-ray diffraction analysis (SC-XRD) ... 8
Crystallographic data ... 9
UV-Vis absorption and emission spectroscopy ... 11
Powder X-ray Diffraction analysis (PXRD) ... 11
Electron diffraction (ED) analysis ... 13
Karl Fischer analysis ... 19
Thermogravimetric Analysis (TGA) ... 20
Variable-temperature Powder X-ray Diffraction Analysis (VT-PXRD) ... 21
Attenuated total Reflection (ATR) Fourier Transform Infrared Spectroscopy (FTIR) analysis. ... 24
ssNMR ... 26
References ... 27
Author Contributions ... 28

Experimental Procedures

Solvents and starting materials were purchased from Merck, Carlo Erba or TCI and used without further purification. All aqueous solutions were prepared from ultrapure laboratory grade water (18 MΩ·cm) obtained from Millipore/MilliQ purification system. NMR spectra were recorded at 9.4T on a Bruker Avance Neo 400 spectrometer. Chemical shifts are reported in ppm with the protic impurities of the deuterated solvent as internal reference. Mass spectra (MS) were obtained with a Thermo Finningan LCQ-Deca XP-PLUS ion trap spectrometer equipped with an electrospray source. HRMS were registered on a ThermoScientific Q-Exactive Plus spectrometer and on a Agilent, Q-ToF G6545B. Water content was determined by coulometric titration with a Metrohm 899 Coulometer.

Preparation of FSTz

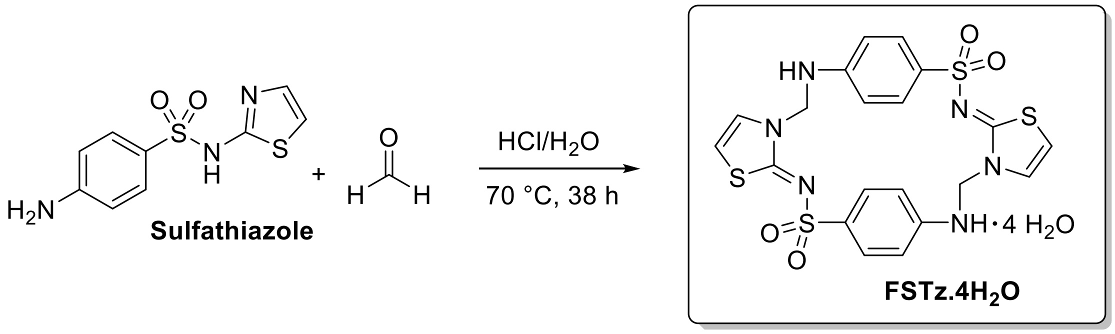

Scheme S1. Synthetic preparation of **FSTz·4H$_2$O**

Formosulfathiazole tetrahydrate (FSTz·4H$_2$O). Sulfathiazole (**Stz**, 10.0 g, 39.2 mmol) was suspended in water (50 mL) and stirred at room temperature. Aqueous 37 % formaldehyde (3.2 mL, 43.1 mmol) was added, and the suspension was heated at 65-70 °C for 22 h. The mixture was cooled to room temperature, filtered under vacuum and the solid was washed with water (15 mL) and dried in a vacuum oven at 55-57 °C until constant weight (typically 16 hours). The crude solid thus obtained (11.0 g) was suspended in water (50 mL) and aqueous 37 % hydrochloric acid (50 µL, 0.604 mmol) was added with stirring. This mixture was heated at 70 °C for 16 h and then cooled to room temperature. The product was isolated by filtration under vacuum and the solid was washed with water (15 mL) and dried in a vacuum oven at 55-57 °C to constant weight (typically 16 hours), obtaining **FSTz·4H$_2$O** (10.3 g, 87%) as a white powder. (Scheme S1)

HPLC chromatogram

HPLC method:

Instrument: YL9300 HPLC system equipped with a variable volume automatic injector
Column: Zorbax SB-CN (4,6x250x5 µm)
Mobile Phase A (MP A): Buffer K_2HPO_4 (pH 7.8, 10 mM)
Mobile Phase B (MP B): Acetonitrile
Mobile Phase C (MP C): Methanol
Column temperature: 10°C
Detection: 254 nm
Flow rate: 0.8 mL/min
Injection volume: 5 µL
Gradient:

Time (min)	% MP A	% MP B	% MP C
0	90	5	5
20	60	12	28
35	60	12	28
37	90	5	5
40	90	5	5

Diluent: DMSO

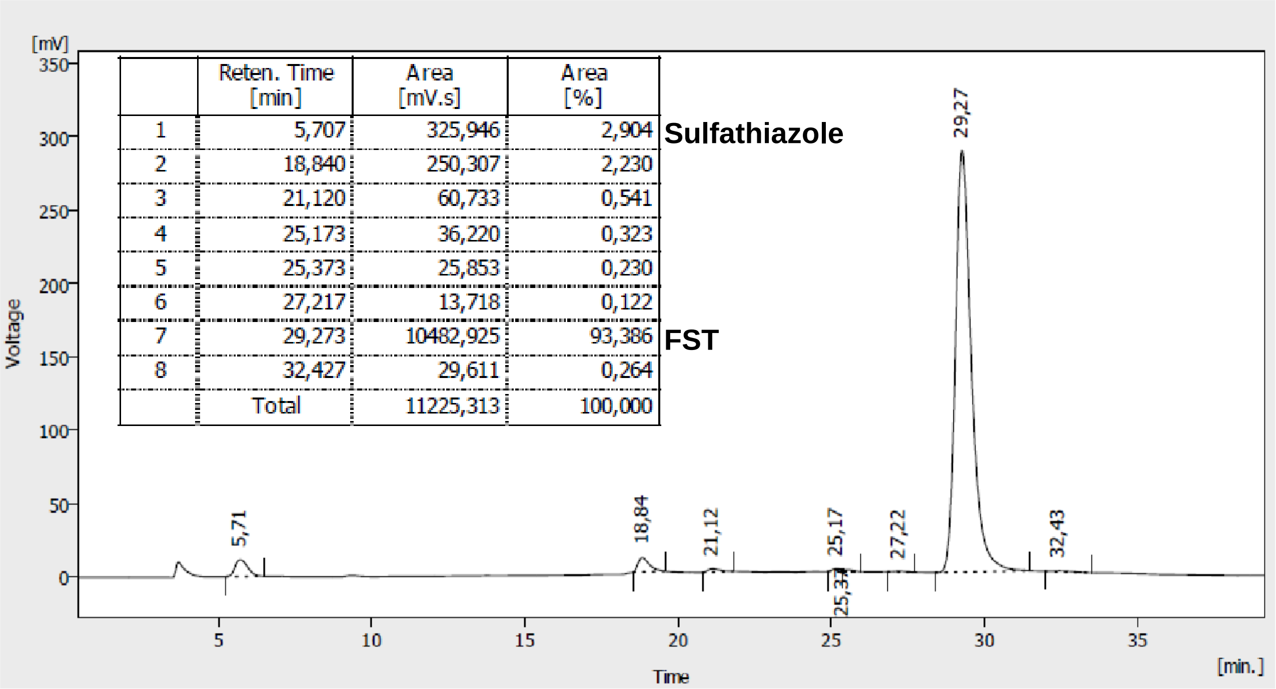

	Reten. Time [min]	Area [mV.s]	Area [%]
1	5,707	325,946	2,904
2	18,840	250,307	2,230
3	21,120	60,733	0,541
4	25,173	36,220	0,323
5	25,373	25,853	0,230
6	27,217	13,718	0,122
7	29,273	10482,925	93,386
8	32,427	29,611	0,264
	Total	11225,313	100,000

Figure S1. HPLC chromatogram of **FSTz**.

Full assignment of ls-NMR ^{1}H and ^{13}C signals of FSTz

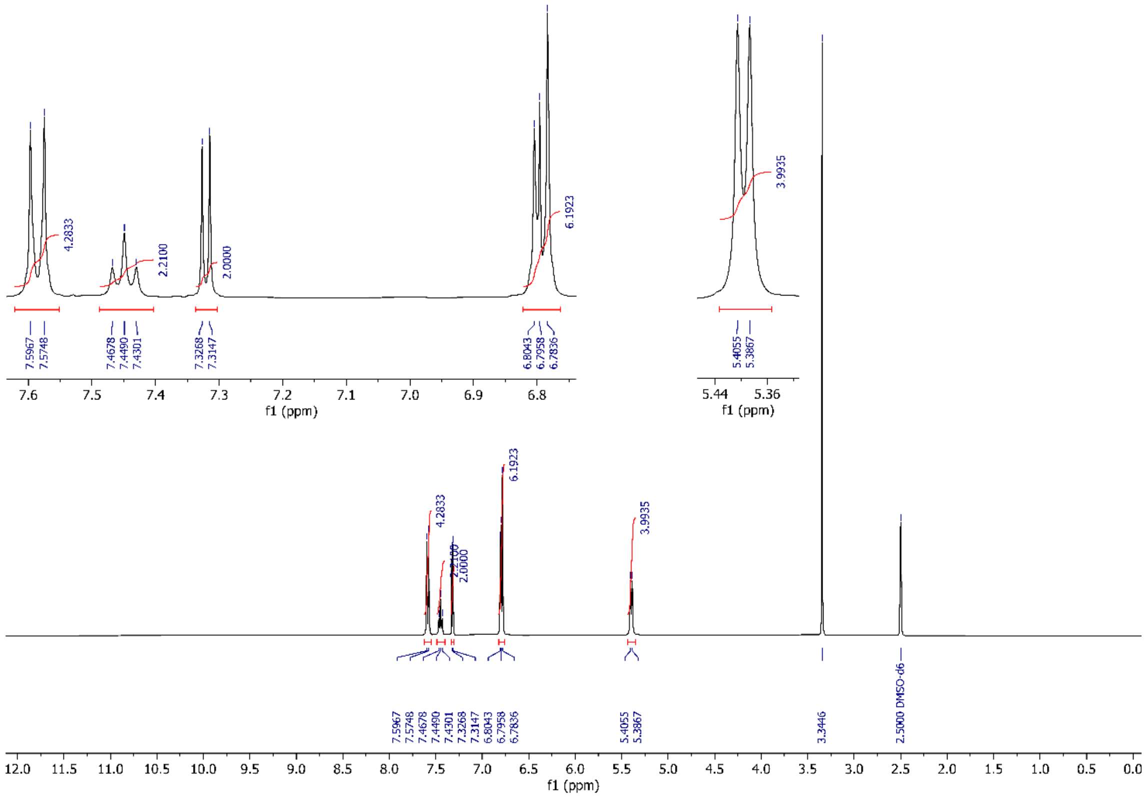

Figure S2. ^{1}H NMR spectrum of **FSTz** (9.4T, 298K, DMSO-d_6).

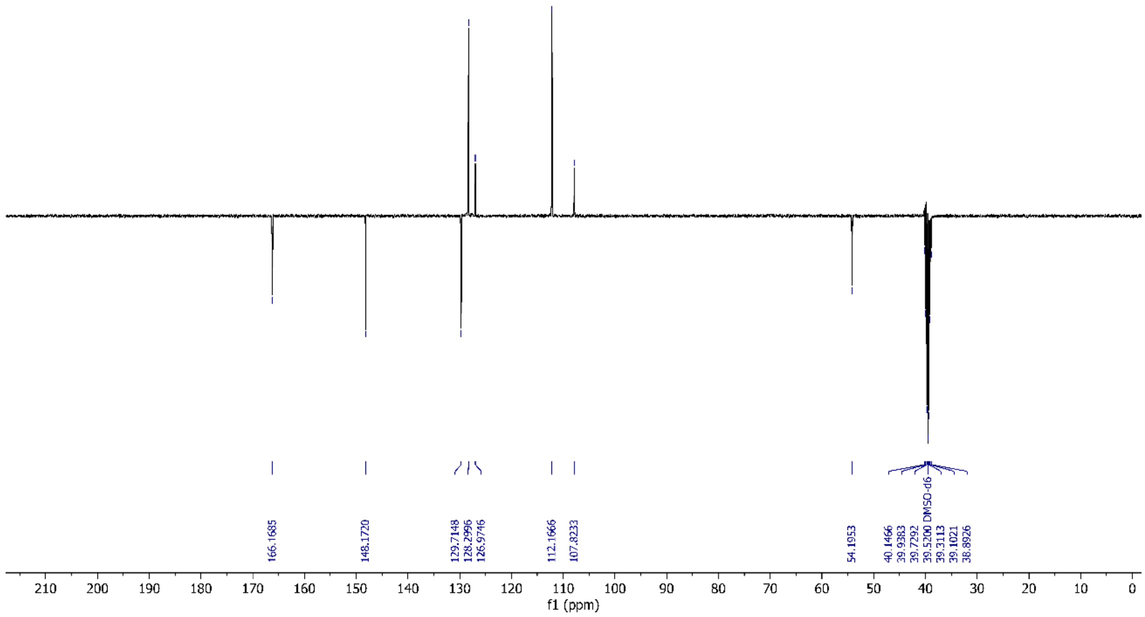

Figure S3. ^{13}C APT NMR spectrum of **FSTz** (9.4T, 298K, DMSO-d_6).

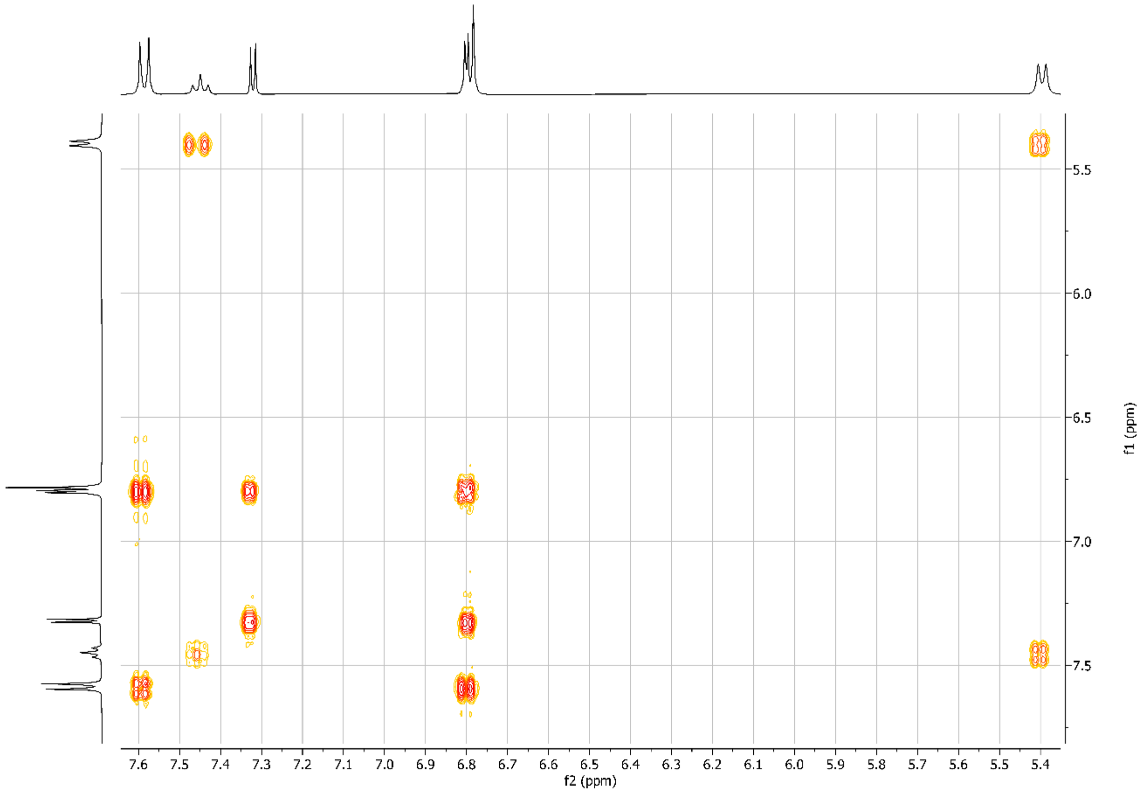

Figure S4. ^{1}H – ^{1}H COSY spectrum of **FSTz** (9.4T, 298K, DMSO-d_6).

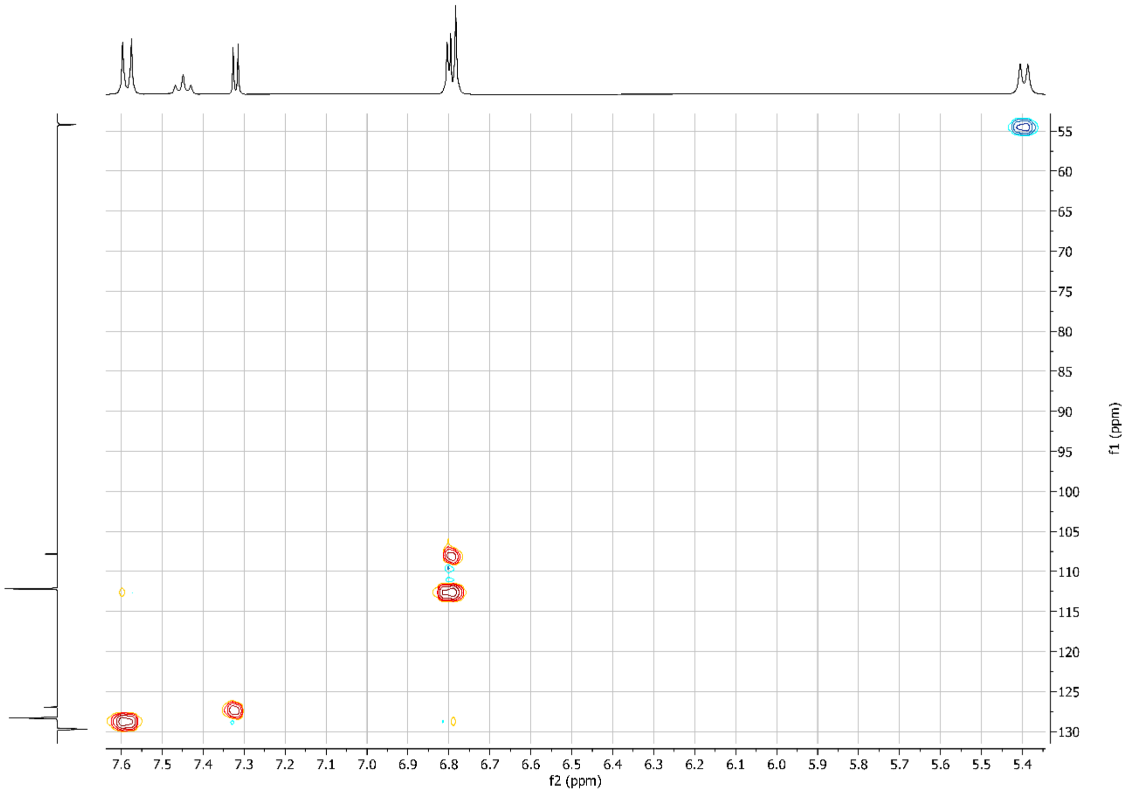

Figure S5. ^{1}H – ^{13}C HSQC spectrum of **FSTz** (9.4T, 298K, DMSO-d_6).

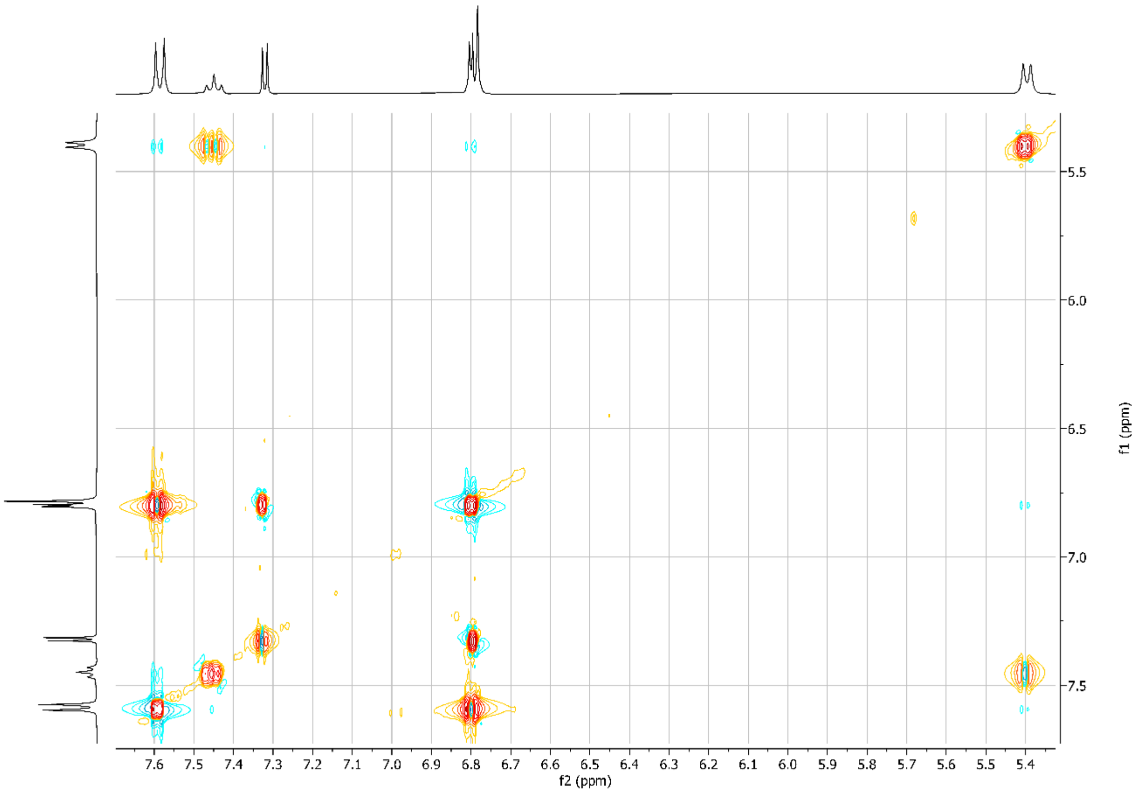

Figure S6. ^{1}H – ^{1}H TOCSY spectrum of **FSTz** (9.4T, 298K, DMSO-d_6).

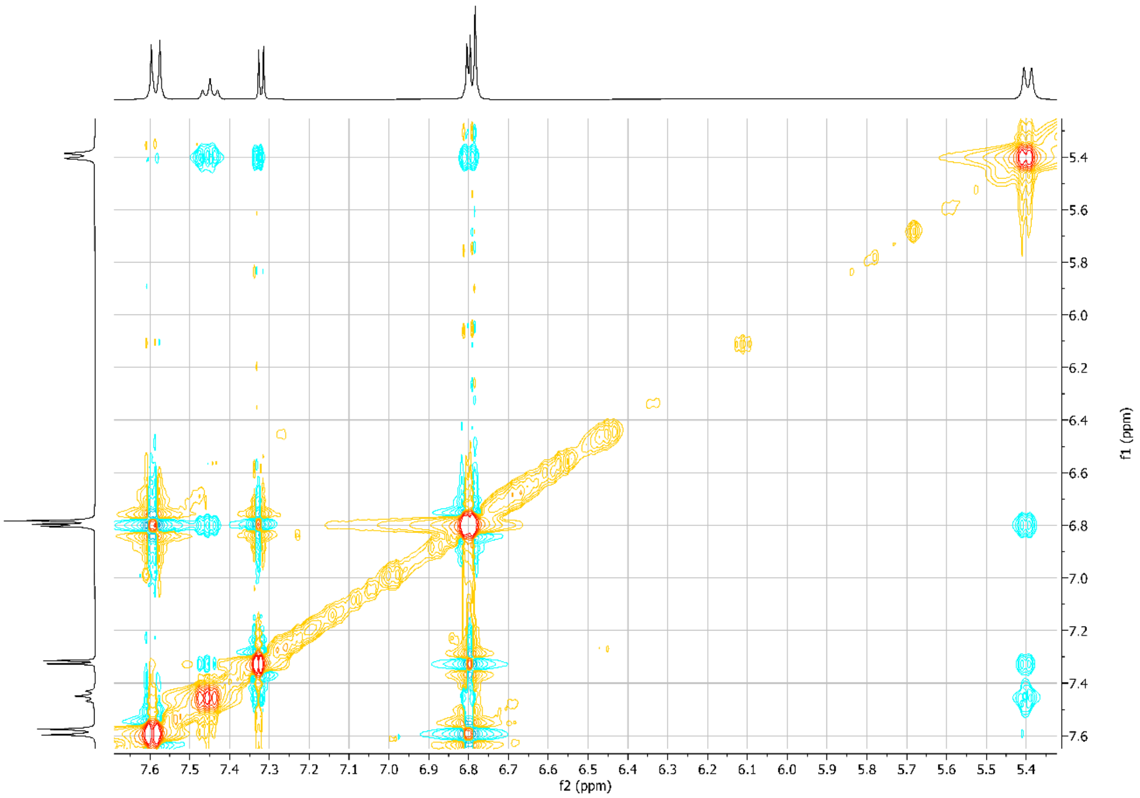

Figure S7. ^{1}H – ^{1}H NOESY spectrum of **FSTz** (9.4T, 298K, DMSO-d_6).

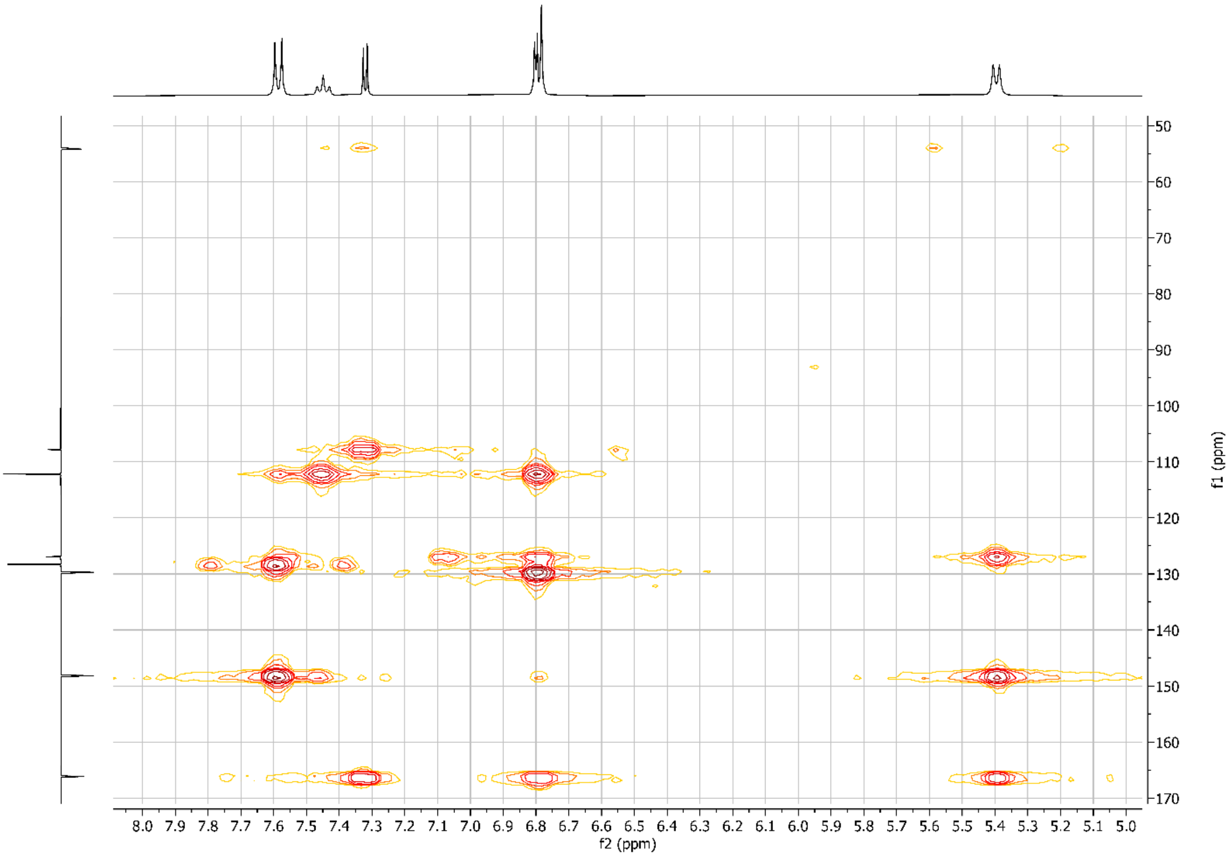

Figure S8. ^{1}H – ^{13}C HMBC spectrum of **FSTz** (9.4T, 298K, DMSO-d_6).

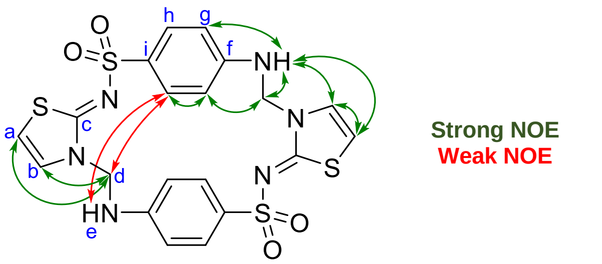

Atom	Type	Proton	^{1}H Peak shape	Carbon
a	CH	6.79	m, 6 H	107.8
b	CH	7.32	d, *J* = 4.8 Hz, 2 H	127.0
c	C	-	-	166.2
d	CH_2	5.40	d, *J* = 7.5 Hz, 4 H	54.2
e	NH	7.45	d, *J* = 7.5 Hz, 4 H	-
f	C	-	-	129.7
g	CH	6.80	m, 6 H	112.2
h	CH	7.59	d, *J* = 8.8 Hz, 4 H	128.3
i	C	-	-	148.2

Table S1. Full assignment of ^{1}H and ^{13}C NMR signals of FSTz and NOE correlations.

^{1}H NMR (400.2 MHz, DMSO-d_6, 298 K) *δ* 7.59 (d, *J* = 8.7 Hz, 4H), 7.45 (t, *J* = 7.5 Hz, 2H), 7.32 (d, *J* = 4.8 Hz, 2H), 6.82 – 6.76 (m, 6H), 5.40 (d, *J* = 7.5 Hz, 4H) ppm.
^{13}C NMR (100.6 MHz, DMSO-d_6, 298 K) *δ* 166.2 (C), 148.2 (C), 129.7 (C), 128.3 (CH), 127.0 (CH), 112.2 (CH), 107.8 (CH), 54.2 (CH_2) ppm.

HRMS analysis

FSTz powder, dissolved in DMSO (100 µg/mL), was analyzed by high-resolution mass spectrometry (Agilent Q-ToF 6545). The [M+H]+ ion at 535.0339 m/z (Δmass = 1.1 ppm) confirmed the molecular formula $C_{20}H_{18}N_6O_4S_4$. The mass spectrum highlights the presence of two species originating in the ion source. The first signal is attributed to the in-source fragmentation of the molecule, leading to the formation of the monomeric unit $[(M/2)+H]^+$ (m/z 268.0201, Δmass = 5.0 ppm). The second signal is related to the in-source formation of the adduct, $[M+(M/2)+H]^+$ (m/z 802.0482, Δmass = 0.6 ppm).

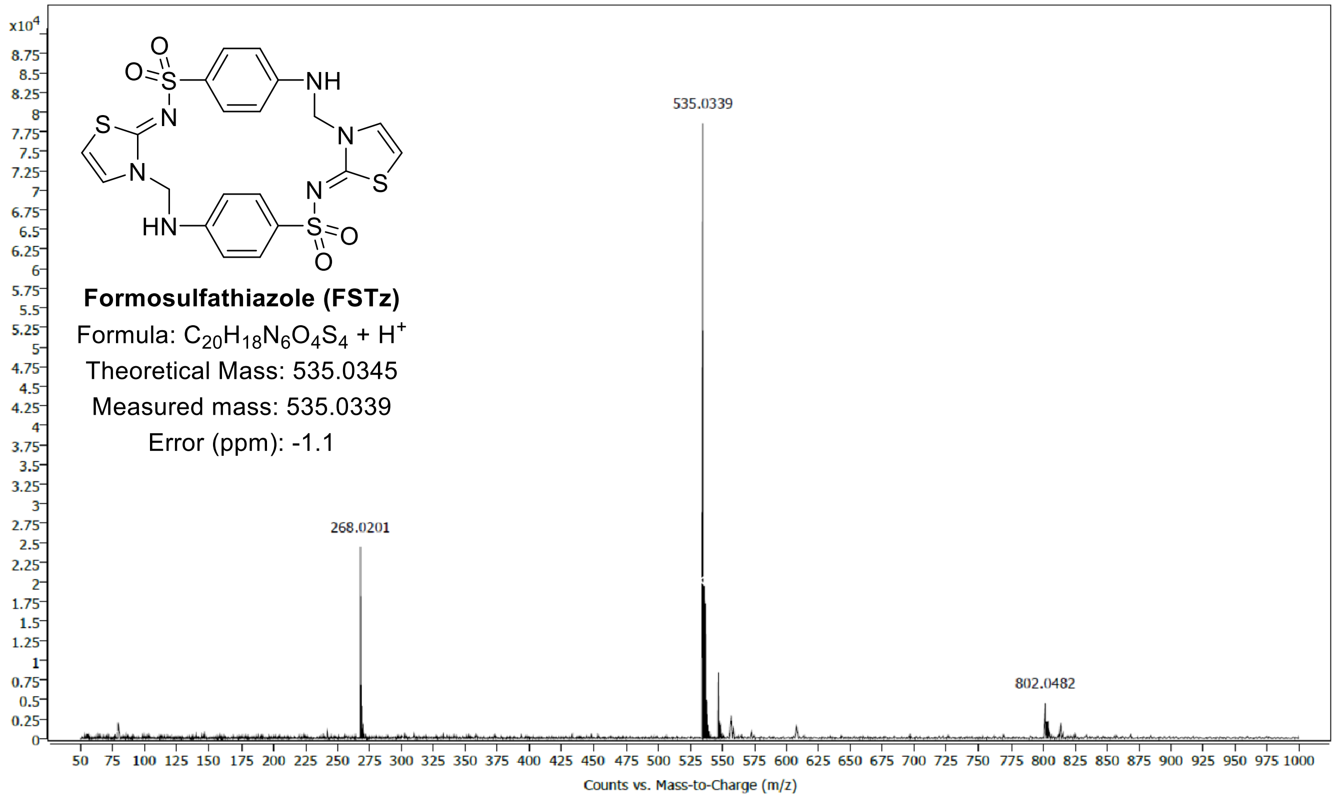

Figure S9. HRMS spectrum of **FSTz**.

Single crystal X-ray diffraction analysis (SC-XRD)

Single crystal X-ray diffraction (SC-XRD) data were collected with an XtaLAB Synergy diffractometer (RIGAKU). The radiation source was a copper micro-focus sealed X-ray tube anode (Cu-Kα, λ = 1.54184 Å) with the generator working at 50 kV and 1 mA. The data reduction was carried out with CrysAlis Pro[1] version 1.171.42.90a using an empirical absorption correction with spherical harmonics (SCALE3 ABSPACK). The structure was solved by dual space methods with SHELXT-2017[2] and refined with SHELXL-2018[3] using the WinGX program suite.[4] Structure refinement was done using full-matrix least-square routines against F^2. All hydrogen atoms on carbon were calculated on idealized positions. Hydrogen atoms connected to heteroatoms were located as residual electron density peaks. Their positions were refined using a riding model.

CCDC 2444187 (FSTz•2DMSO) and CCDC 2430519 (FSTz·4H_2O) contain the supplementary crystallographic data for this paper. These data and additional information can be obtained free of charge via https://summary.ccdc.cam.ac.uk/structure-summary-form (or from the Cambridge Crystallographic Data Centre, 12 Union Road, Cambridge CB2 1EZ, UK; fax: (+44)1223-336-033; or deposit@ccdc.cam.uk).

FSTz·2DMSO crystallizes in the space group $P2_1/n$ with one a cyclodimeric structure of FSTz and two molecules of DMSO all lying in general position (Z = 4, Z' = 1). The molecular subunits of sulphathiazole are linked head-to-tail by the methylene groups bridging the amine group on the benzene ring with the endocyclic nitrogen atom of the thiazole ring, the latter being found in the non-aromatic 2-iminothiazoline tautomeric form. The dimer has an approximate C_2 symmetry. Intermolecular N–H···O hydrogen bonds are observed between the cyclodimeric ring and the DMSO molecule, with distances of N3(-H)···O5 = 2.846(2) Å and N6(-H)···O6 = 2.897(2) Å. Additionally, on both sides of the cyclodimeric ring, a symmetric set of intra- and intermolecular interactions occurs between the sulfur atoms of the thiazole rings and the oxygen atoms of the sulfonic groups. Specifically, each sulfur atom in the thiazole rings engages in intermolecular interactions with the oxygen of the adjacent sulfonic group (S2···O2 = 2.882 Å and S4···O4 = 2.876 Å), as well as with a second oxygen from the sulfonic group of another ring (S2···O3 = 3.110 Å and S4···O1 = 2.991 Å).

Crystallographic data

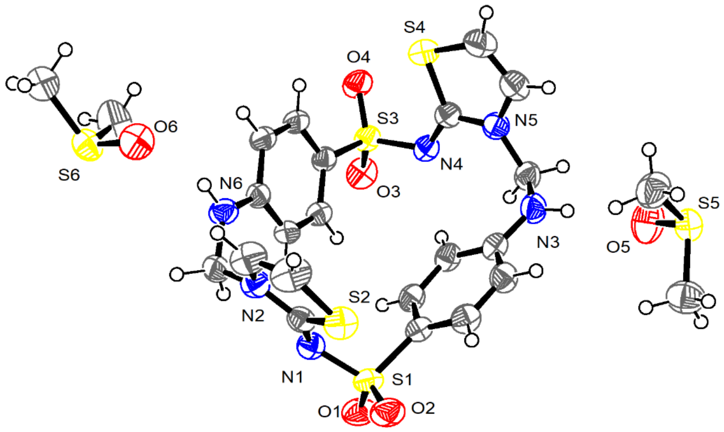

Figure S10. Ortep representation of **FSTz·2DMSO** asymmetric unit with labels. Same labels have been used for all crystal structures reported in this manuscript. Colour code: C, grey; N, blue; O, red; S, yellow; H, white. Ellipsoids are drawn at 50% probability.

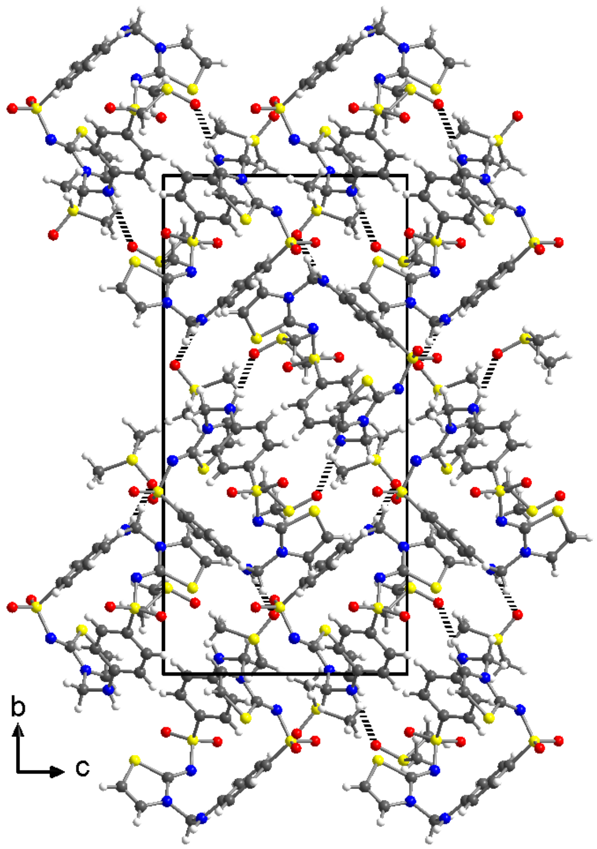

Figure S11. Crystal packing of **FSTz·2DMSO** viewed along the crystallographic [100] direction. Hydrogen bonds of the type N(-H)···O are depicted with dashed black lines. Colour code: C, grey; N, blue; O, red; S, yellow; H, white.

**Chemical compound**	**Interaction type**<br>**D-H···A**	**Distance / Å**<br>**D(-H) ··· A**	**Angle / °**<br>**D-H-A**
**FSTz·2DMSO**	N6-H4 ··· O6<br>N3-H1 ··· O5	2.897(2)<br>2.846(2)	161(2)<br>175(3)

Table S2. Hydrogen bond intermolecular interactions observed in FSTz·2DMSO.

Refcode	Crystalline form of sulfathiazole in its tautomeric form	Refcode	Crystalline form of sulfathiazole in its tautomeric form
ADEDIX(01-02)	Pyridine solvate	KAWZER	α-Methyl-γ-butyrolactone solvate
BABYIN	Acetonitrile clathrate	KAWZIV	Diethyl oxalate solvate
BABYOT	*N*-Formylpiperidine solvate	KAWZOB	Sulfolane solvate
BUWDUT	Salt of hexafluorosilicic acid	KAWZUH	Methylisoxazole solvate
DOVGAA	Pyrimethaminium sulfathiazolate sulfathiazole ethanol-solvated salt cocrystal	KAXNUW	Salt of trimethoprim and pimelic acid
DOVGEE	Pyrimethaminium sulfathiazolate sulfathiazole isopropanol-solvated salt cocrystal	KAXPIM	Acetone solvate monohydrate salt of trimethoprim and sebacic acid
DOVGII	Pyrimethaminium sulfathiazolate sulfathiazole acetonitrile-solvated salt cocrystal	KUFWIT	Salt of 2,4-dinitrobenzoic acid
DOVGOO	Pyrimethamine sulfathiazole cocrystal	KUFWOZ	4-Aminobenzamide cocrystal
FIZFUR	4-Nitrobenzoic acid cocrystal	LOFLUP	Glutaric acid cocrystal
GEKKIU	*N*-Methylacetamide cocrystal	LOFMAW(01)	Salt of oxalic acid
HADMUU	(18-crown-6) acetonitrile clathrate	OXIDEG02	4,4'-bipyridine 1,1'-dioxide cocrystal
JUVFOY(01)	Pyridin-2-amine cocrystal	PASLOO	Hemihydrate salt of pyridine-2,6-dicarboxylic acid
JUVFUE	2-Amino-4-methylpyridine cocrystal	PASMAB	Hydrate salt of a complex of zinc with pyridine-2,6-dicarboxylic acid and resorcinol
JUVGAL(01)	2-Amino-4-chloropyridine cocrystal	PASMEF	Hydrate salt of a complex of zinc with pyridine-2,6-dicarboxylic acid and 3-nitrophenol
JUVGEP	2-Amino-4-bromopyridine	SULTHE01	Theophylline cocrystal
KAWMEE	Cyclopentanone solvate	SUTHAZ(01-02,05-06, 15-50)	Polymorphs
KAWMII	4-methylthiazole solvate	TAPCIA	2-Amino-3-nitropyridine cocrystal
KAWMOO	Propionitrile solvate	TAPCUM	2-Amino-5-nitropyridine cocrystal
KAWMUU	Cyclohexanol solvate	TAPDEX	4,4'-Bipyridine cocrystal
KAWNAB	Cyclohexanecarbonitrile solvate	TAPDIB	Salt of 3,5-dinitrobenzoic acid
KAWPOR	(-)-Sparteinium sulfathiazolate	UDAKOA	Monohydrate salt of nitric acid
KAWQIM	Pyrrolidine-1-carbonitrile solvate	VEYQUO	Salt of nitric acid
KAWQOS	5-Methyl-2(3*H*)-furanone solvate	VEYRAV	Salt of tetrafluoroboric acid
KAWQUY	Bis(caprolactam) cocrystal	VEYREZ	Monohydrate salt of sulfuric acid
KAWRAF	Beta-butyrolactone solvate	VEYYEG	Salt of benzensulfonic acid
KAWREJ	(+/-)-2-methylcyclopentanone solvate	VUKTAZ	Amantadine hydrochloride cocrystal
KAWRIN	Cyclohexanone solvate	WIYLAT	2,4,6-Tris(pyridin-2-yl)-1,3,5-triazine cocrystal
KAWROT	Cycloheptanone solvate	XEHTOX	1,12-dodecanedinitrile solvate
KAWRUZ	Gamma-valerolactone solvate	XEHTUD	Triethyl phosphate solvate
KAWSAG	Gamma-butyrolactone solvate	XEHVAL	Butyronitrile solvate
KAWSEK	Cyclo-octanone cocrystal	XEHVEP	Valeronitrile solvate
KAWSIO	4-Methylcyclohexanone solvate	XEHVIT	Morpholine-4-carbonitrile solvate
KAWSOU	*N,N'*-dimethylpropylene urea solvate	XEHVOZ	Azelanitrile solvate
KAWSUA	Cyclopentanol solvate	XEHVUF	1,6-Hexanedinitrile solvate
KAWTAH	Tetrahydrofuran solvate	XEHWAM	Pyridazine solvate
KAWTEL	4-Methyl-1,3-dioxalene-2-one solvate	XEHWEQ	2,4,8,10-Tetraoxaspiro[5.5]undecane cocrystal
KAWTIP	1,3 Dimethyl-2-imidazolidone solvate	XEHWIU	δ-Valerolactone solvate
KAWTOV	1,8-Octanedinitrile solvate	XEHWOA	Acetone solvate
KAWTUB	1,10-Decanedinitrile solvate	XEHWUG	Cyclopropanecarbonitrile solvate
KAWYUG	Diethyl malonate solvate	YIXMIE	Salt of a tetrachloro-cobalt complex and acetic acid
KAWZAN	*N,N*-Dibutylformamide solvate	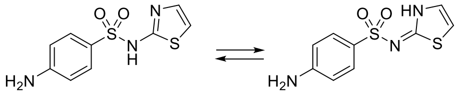 **Scheme S2.** Sulfathiazole and its tautomeric form.	

Table S3. List of polymorphs, cocrystals and salts of sulfathiazole (STz) in its tautomeric form found in the Cambridge Structural Database (CSD).[1] The CSD identifier (refcode) is reported in the first column while the second column describes the crystalline form of STz.

UV-Vis absorption and emission spectroscopy

UV-Vis absorption spectra of **FSTz** and **STz** dissolved in DMSO were recorded in the 200-600 nm range (1 nm interval) using a double beam Shimadzu UV1900 UV-vis spectrophotometer (Shimadzu Italia, Milan, Italy)
Fluorescence spectra of **FSTz** and **STz** dissolved in DMSO were acquired at 25 °C on a Jasco FP8500 instrument (Jasco Europe, Cremella, Italy). The excitation wavelength was set to 288 nm and spectra were acquired in the 300-550 nm range (data interval 1 nm, scan speed 1000 nm/min) with excitation bandwidth set at 5 nm and emission bandwidth set at 10 nm.

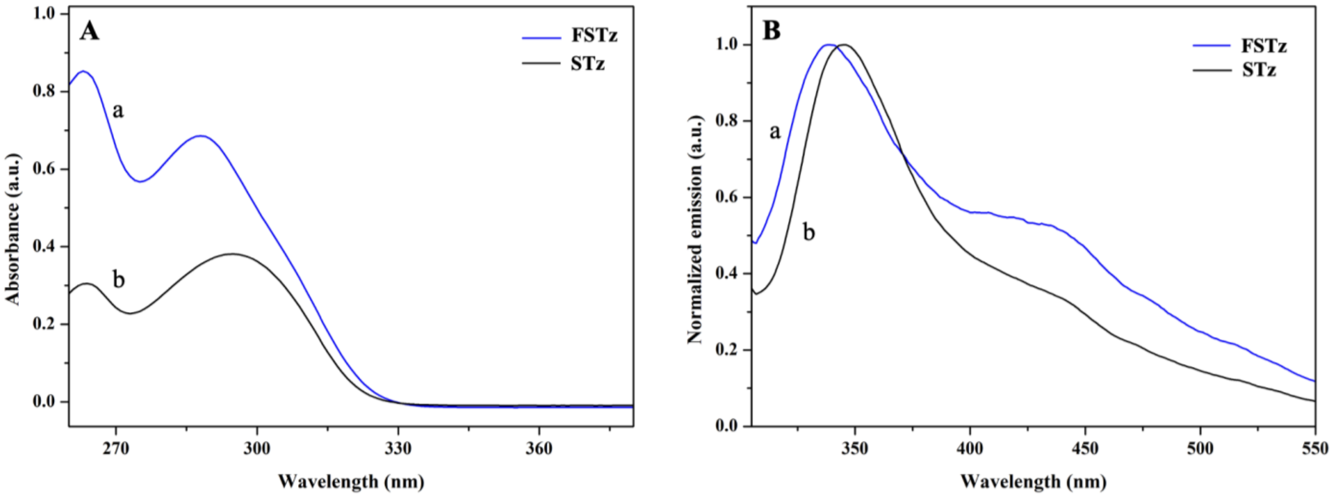

Figure S12. UV absorption spectra (A) and fluorescence spectra (B) of formosulfathiazole (**FSTz**, curve a) and sulfathiazole (**STz**, curve b).

Powder X-Ray Diffraction analysis (PXRD)

PXRD patterns were collected using Cu-Kα radiation (λ=1.5418 Å) on a vertical-scan Bruker AXS D8 Advance diffractometer in θ:θ mode, equipped with a Goebel Mirror and a Bruker Lynxeye Linear Position Sensitive detector, with the following optics: primary and secondary Soller slits, 2.3° and 2.5°, respectively; divergence slit, 0.1°; receiving slit, 2.82°, generator setting: 40 kV, 40 mA. The nominal resolution for the present set-up is 0.08° 2θ (FWHM of the α 1 component) for the LaB_6 peak at about 21.3° (2θ).
The accurate diffraction patterns at RT of FSTz was acquired in the 5-90 2θ range, with Δ2θ = 0.02° and exposure time 5 s/step. The structural process for the Rietveld refinement is here described in detail: a standard peak search below 30° 2θ was followed by indexing through the singular value decomposition method,[5] implemented in TOPAS-Academic 6,[6,7] which led to a monoclinic cell of approximate dimensions: a = 27.847 Å, b = 11.151 Å, c = 17.612 Å, β = 97.54°, and V = 5422.33 Å³ (GoF(20) = 31.33). Systematic absences and volume considerations led to individuate *C*2/*c* as the most probable space group. A Le Bail refinement was carried out (a = 27.8416(5) Å, b = 11.1493(2) Å, c = 17.6113(4) Å, β = 97.522(2)°, and V = 5419.7(2) Å³, R_{wp} 3.958 %, Figure S14) in order to determine the background, cell and profile parameters to be used in the subsequent runs.[8] The preliminary work done with indexing and structureless le Bail refinement confirmed the purity of the sample. As described in the main text, Rietveld model was determined starting from the ED results. Rigid bodies were used to describe the FSTz dimer and 4 water molecules. The position of center of mass and molecular orientation for each rigid body were let to refine. The peak shapes were described with the fundamental parameters approach.[9] The background was modelled by a Chebyshev polynomial function. The thermal effect was simulated by using a single isotropic parameter for all atoms. The final Rietveld refinement plot is supplied in Fig. S21. Final figures of merit *R*p: 7.194 %; *R*wp: 9.368 %; *R*bragg: 5.769 %, for the data in the 3-90 2θ range (λ = Cu-Kα).

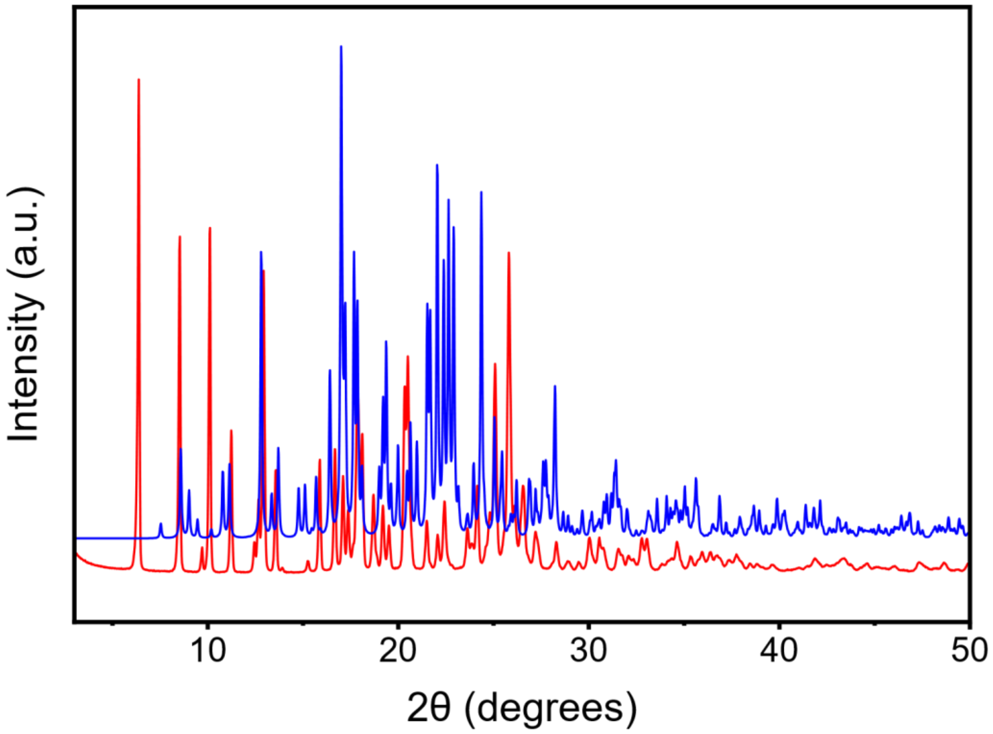

Figure S13. Comparison between the experimental pattern of the bulk sample (red trace) and the calculated powder pattern for the **FSTz·2DMSO** crystallographic structure determined from SC-XRD data (blue trace).

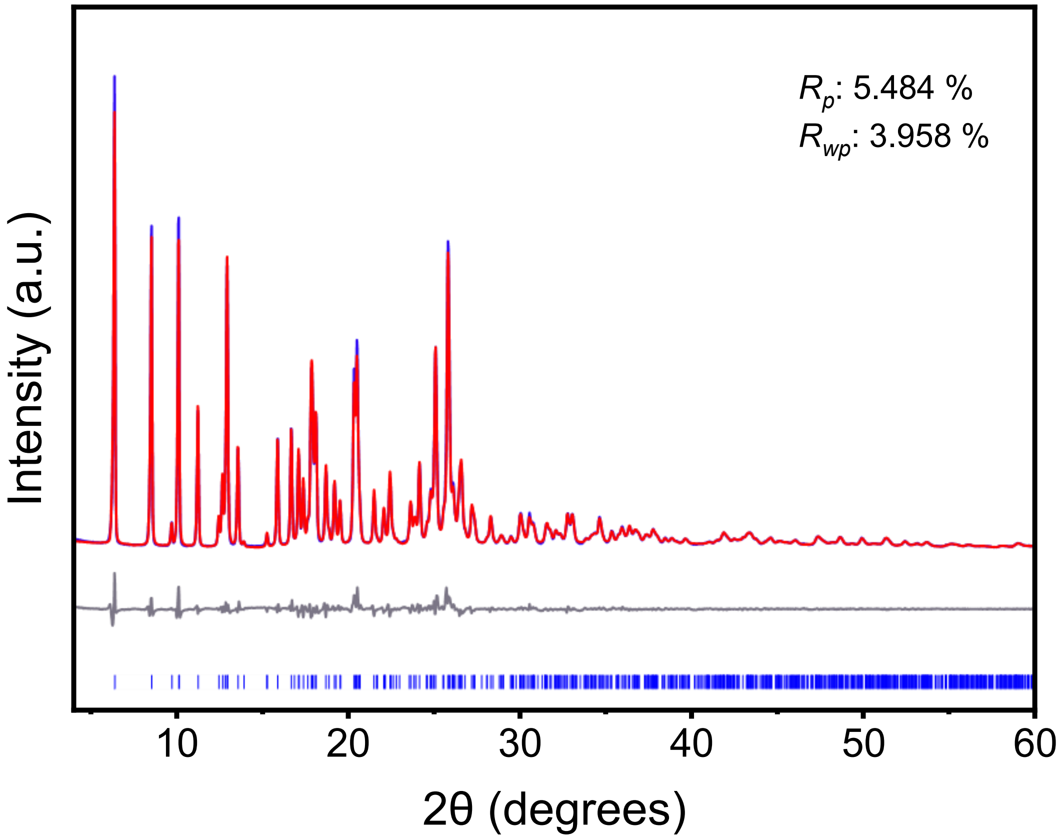

Figure S14. Structureless Le Bail fitting plot for **FSTz·4H$_2$O**. Blue trace and red trace represent the experimental and calculated diffractograms, respectively. Grey line represents the difference between the experimental and calculated profiles. Blue tick marks are the calculated peak positions. *R*p and *R*wp, 5.484 % and 3.958 % respectively, for the data in the 3-60 2θ range (λ = Cu-Kα). Space group, *C*2/*c*; structural parameters refined on the basis of the indexed proposed solution *a* = 27.8416(5) Å, *b* = 11.1493(2) Å, *c* = 17.6113(4) Å, *β* = 97.522(2)°, and *V* = 5419.7(2) Å^3.

Electron diffraction (ED) analysis

Continuous rotation 3D electron diffraction data of **FSTz·4H$_2$O** were acquired using the dedicated electron diffractometer Rigaku XtaLAB Synergy-ED, equipped with a HyPix-ED detector by Rigaku Oxford Diffraction. Data acquisition was performed at 100 K using a Gatan Elsa cryotransfer holder with an electron wavelength of 0.0251 Å (200 kV). The data were processed using CrysAlisPro 1.171.44.92a, the structure was solved using ShelXT 2018/3 and subsequently refined using the kinematical approximation with ShelXL 2018/2 in the crystallographic program suite Olex2 1.5-ac7-013. Hydrogen atoms were placed on geometrically restrained positions using bond distances obtained by neutron diffraction.[10] By merging the data of three individual crystals, a completeness of 99.9% up to a resolution of 0.80 Å was achieved. Key crystallographic and metric data are summarized in Tables S5 and S6, respectively. The crystals screened for the diffraction analysis can be seen in Figure S15.

Grain No.	Crystal size [µm]	Temperature [K]	Scan range [°]	Scan width [°]	Exposure time/frame [s]	Dose [e^-/(Å^2)]
1	1.2 x 0.7 x 0.3	100.0	-57 to 59	0.25	0.5	1.09
2	0.8 x 0.6 x 0.3	100.0	-59 to 61	0.25	0.5	1.12
3	1.1 x 0.7 x 0.3	100.0	-61 to 64	0.25	0.5	1.17

Table S4. Experimental conditions for 3D-ED measurements.

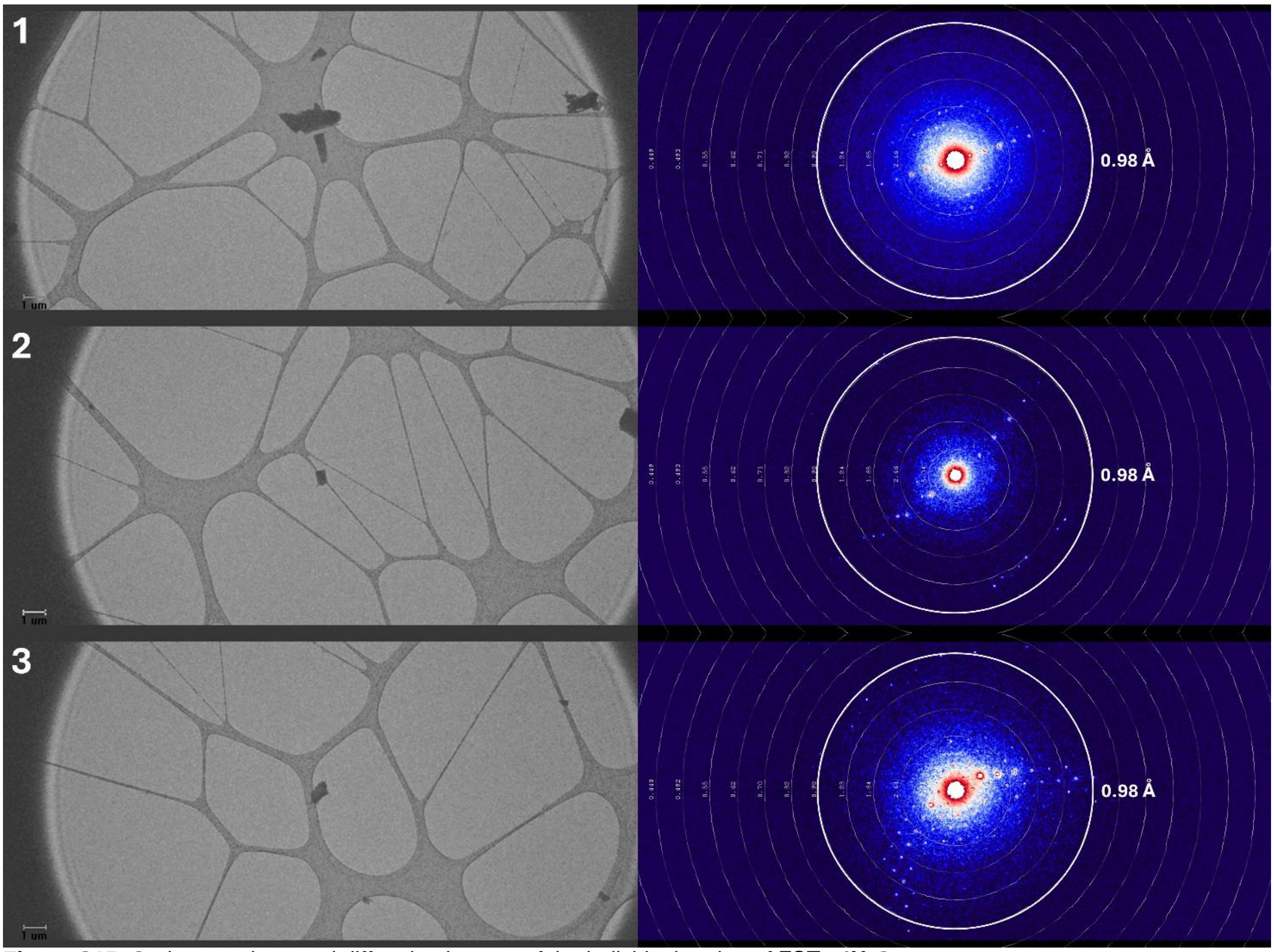

Figure S15. Grain snapshots and diffraction images of the individual grains of **FSTz·4H$_2$O**.

Atom	Atom	Length/Å
S3	O3	1.436(8)
S3	N4	1.611(8)
S3	O4	1.430(9)
S3	C8	1.755(9)
S4	C11	1.746(10)
S4	C12	1.735(10)
S1	O2	1.446(10)
S1	O1	1.445(11)
S1	N1	1.596(10)
S1	C18	1.739(10)
S2	C1	1.755(12)
S2	C2	1.739(13)
N6	C15	1.379(11)
N6	C14	1.424(11)
N4	C11	1.335(11)
C16	C15	1.418(11)
C16	C17	1.404(12)
C11	N5	1.354(11)
C15	C20	1.385(11)
C20	C19	1.371(11)
N5	C14	1.499(12)
N5	C13	1.350(12)
C8	C7	1.388(12)
C8	C9	1.382(12)
C7	C6	1.383(12)
C17	C18	1.404(12)
C5	N3	1.395(12)
C5	C10	1.398(13)
C5	C6	1.425(13)
C9	C10	1.383(12)
N1	C1	1.312(14)
N3	C4	1.437(14)
C19	C18	1.422(12)
N2	C1	1.365(13)
N2	C4	1.484(14)
N2	C3	1.375(15)
C13	C12	1.389(14)
C3	C2	1.335(15)

Atom	Atom	Atom	Angle/˚
O3	S3	N4	105.5(5)
O3	S3	C8	108.5(5)
N4	S3	C8	105.5(4)
O4	S3	O3	116.8(5)
O4	S3	N4	112.7(5)
O4	S3	C8	107.2(5)
C12	S4	C11	91.8(5)
O2	S1	N1	112.8(6)
O2	S1	C18	105.9(5)
O1	S1	O2	115.9(6)
O1	S1	N1	107.5(6)
O1	S1	C18	108.2(5)
N1	S1	C18	106.0(5)
C2	S2	C1	91.4(6)
C15	N6	C14	124.2(7)
C11	N4	S3	118.3(7)
C17	C16	C15	119.7(8)
N4	C11	S4	129.0(7)
N4	C11	N5	121.5(8)
N5	C11	S4	109.5(6)
N6	C15	C16	117.0(7)
N6	C15	C20	123.7(8)
C20	C15	C16	119.2(8)
C19	C20	C15	122.2(8)
C11	N5	C14	123.4(7)
C13	N5	C11	115.4(8)
C13	N5	C14	121.2(7)
N6	C14	N5	112.2(7)
C7	C8	S3	119.3(7)
C9	C8	S3	120.4(7)
C9	C8	C7	120.1(8)
C6	C7	C8	120.4(9)
C16	C17	C18	119.8(8)
N3	C5	C10	123.0(9)
N3	C5	C6	118.8(9)
C10	C5	C6	118.2(8)
C8	C9	C10	120.2(9)
C1	N1	S1	121.2(8)
C5	N3	C4	122.3(9)
C9	C10	C5	120.9(9)
C20	C19	C18	119.2(8)
C17	C18	S1	121.7(7)
C17	C18	C19	119.8(8)
C19	C18	S1	118.5(7)
C1	N2	C4	121.5(9)
C1	N2	C3	116.2(9)
C3	N2	C4	122.2(9)
N5	C13	C12	114.2(8)
N1	C1	S2	128.3(8)
N1	C1	N2	123.8(10)
N2	C1	S2	107.9(8)
C13	C12	S4	109.0(7)
C7	C6	C5	120.0(9)
N3	C4	N2	112.6(9)
C2	C3	N2	113.1(10)
C3	C2	S2	111.4(9)

Table S5. Bond length and bond angles for FSTz·4H_2O

Chemical compound	Interaction type D-H···A	Distance / Å D(-H) ··· A	Angle / ° D-H-A
	N6-H6 ··· O3[1]	2.984(10)	162.2
	C16-H16 ··· O3[1]	3.425(11)	136.9
	C9-H9 ··· O4[2]	3.311(12)	140.1
	N3-H3 ··· O5[3]	3.019(15)	132.0
	C19-H19 ··· O4[2]	3.152(11)	143.3
	C4-H4A ··· O2[4]	3.222(13)	135.9
	C2-H2 ··· O6[4]	3.488(17)	125.1
**FSTz·4H_2O**	O5-H5A ··· O6	2.732(18)	172.8
	O5-H5B ··· O1	2.855(14)	156.5
	O6-H6B ··· O7	2.79(2)	149.6
	O6-H6C ··· O2[5]	2.899(15)	176.2
	O7-H7A ··· O5[6]	2.78(2)	141.4
	O7-H7B ··· N4[7]	3.213(18)	133.7
	O8-H8A ··· O7	2.92(3)	147.9
	O8-H8B ··· N1[6]	3.292(17)	170.6

Table S6. Hydrogen bond intermolecular interactions observed in FSTz·4H_2O.

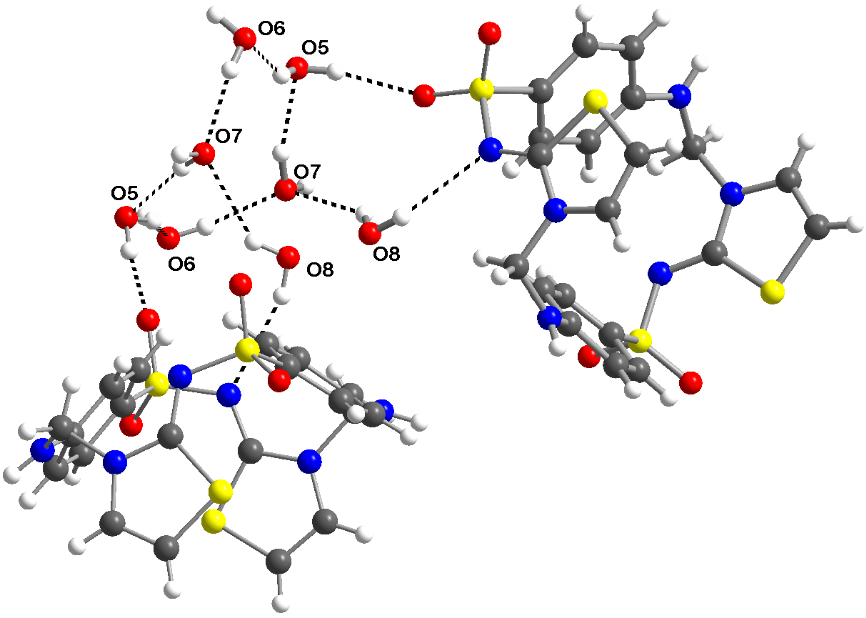

Figure S16. Octameric cluster of H-bonded water molecules with labels highlighting symmetry relationships (C_2 symmetry axis). The water molecules in the cluster are arranged similar to a bicyclo[2.2.2]octane structure, thus stabilizing the reticular energy of the network. Color code: C, grey; N, blue; O, red; S, yellow; H, white. H-bonds are depicted with dashed black lines.

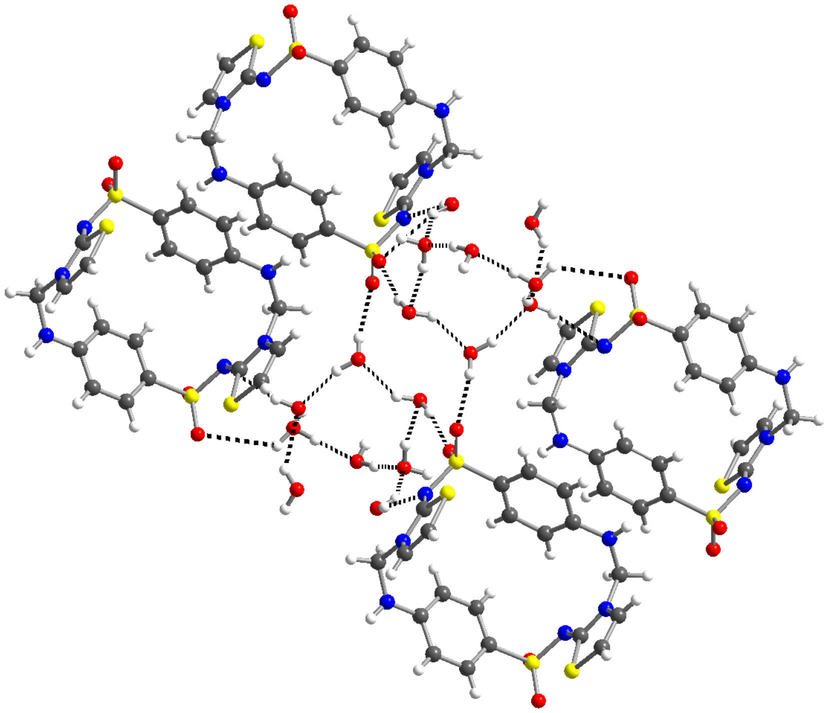

Figure S17. Particular network arrangement of water molecules in **FSTz·4H$_2$O**. Bond distances and angles are reported in Table 6. Color code: C, grey; N, blue; O, red; S, yellow; H, white. H-bonds are depicted with dashed black lines.

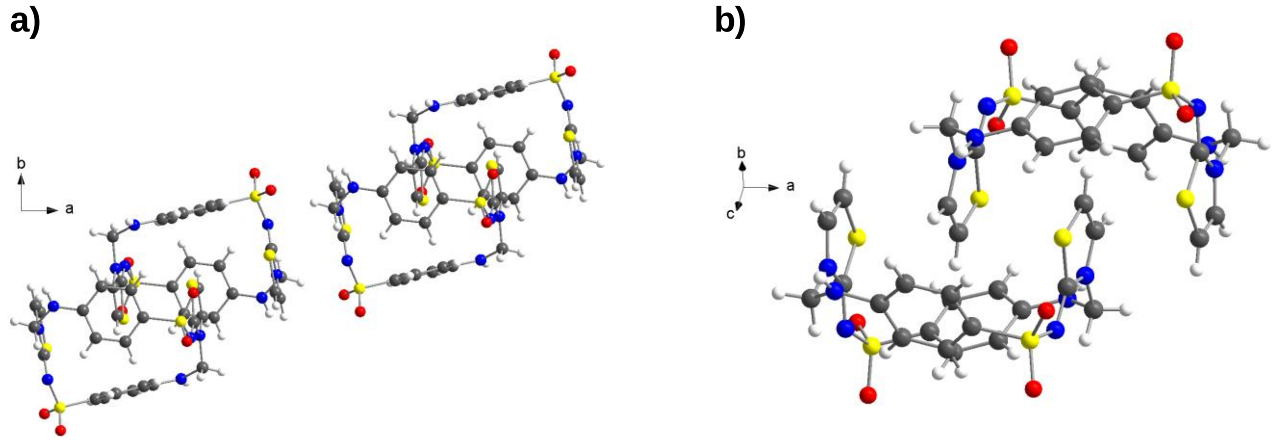

Figure S18. Details of the two cyclodimeric rings arranged in a face-to-face configuration through an inversion center, further stabilizing the network via weak intermolecular interactions between the thiazole rings. (a) Chains of paired **FSTz** rings viewed along the [001] direction. (b) Close-up view showing one ring embracing the other. Color code: C, grey; N, blue; O, red; S, yellow; H, white. H-bonds are depicted with dashed black lines.

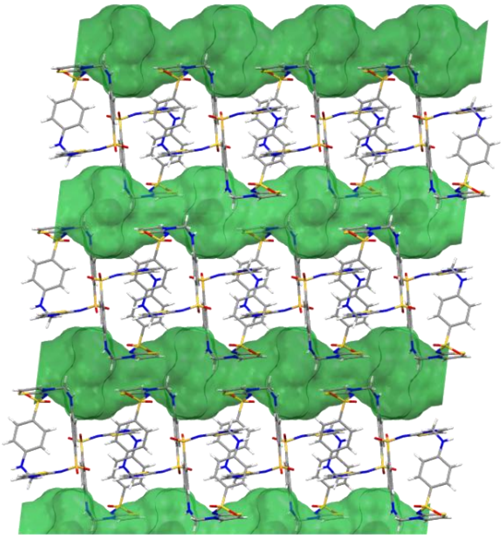

Figure S19. Representation of the crystal packing viewed down the [010] direction in which the crystallization water molecules have been removed, and the empty space left is shown in green. Channels widen and narrow along the crystallographic [001] direction. Voids: 19.8 % (calculated with Mercury 4.0).[11] Color code: C, grey; N, blue; O, red; S, yellow; H, white.

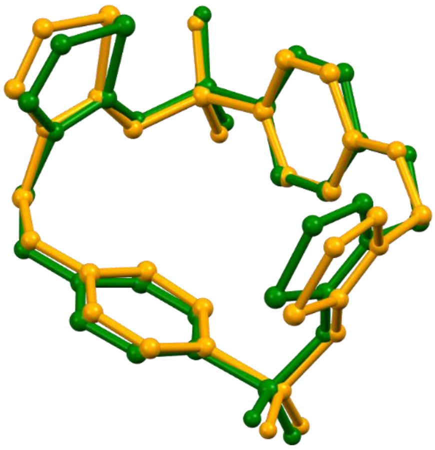

Figure S20. Comparison of the conformation of **FSTz** molecule in **FSTz·2DMSO** and **FSTz·4H₂O** showing them to be almost identical. Color code: **FSTz·2DMSO**, orange; **FSTz·4H₂O**, green.

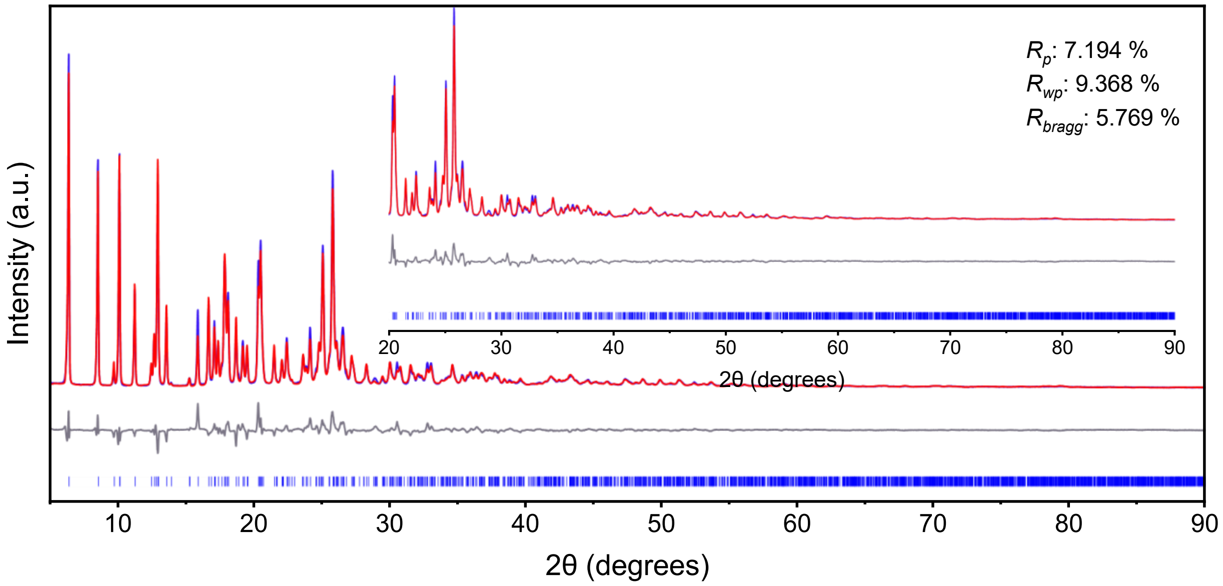

Figure S21. Rietveld refinement for **FSTz·4H₂O**. The blue trace represents the experimental pattern, the red trace the calculated one and the grey line the difference between the experimental and calculated profiles. Blue tick marks are the calculated peak positions. R_p: 7.194 %; R_{wp}: 9.368 %; R_{bragg}: 5.769 %, for the data in the 3-90 2θ range (λ = Cu-Kα). Space group, *C2/c*; a axis, 27.806(1) Å; b axis, 11.1357(5) Å; c axis, 17.5881(8) Å; β angle, 97.542(4) °.

Karl Fischer analysis

Coulometric titration analysis of **FSTz·4H₂O** reveals a water content consistent with the presence of four molecules of water of hydration. The analysis was conducted on a 10 mg/mL solution in *N*-methylpyrrolidone (NMP), and the result was 11.4% water by weight (RSD = 1.4%).

	NMP density (g/mL)
NMP	1.028
N-Methylpyrrolidone, #I1312697, supplied by Merck	

Sample sol

Weight of **FSTz·4H₂O** (mg)	Volume of NMP (mL)	Conc (mg/mL)	Conc (mg/g)
50.11	5	10.022	9.749

Blank (NMP)		
Weight NMP (mg)	mg H_2O (in NMP)	%H_2O in NMP
1078.05	1.9356	0.1795

Analysed Sample sol

Weight Sample sol. (mg)	Weight of **FSTz·4H₂O**	measured mg H_2O	mg H_2O - blank	% H_2O	Mean	SD	% RSD
1058.5	10.3193165	3.0433	1.1618	11.26	11.42 %	0.156	1.37
1022.12	9.96464788	2.9565	1.1397	11.44			
1001.87	9.76723063	2.9112	1.1304	11.57			

Formosulfathiazole

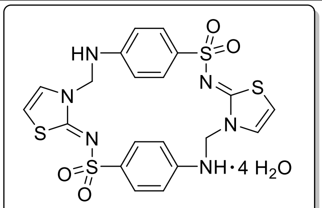

MF	MW
$C_{20}H_{18}N_6O_4S_4 \cdot 4H_2O$	606.73
$4H_2O$	72.06
theoretical analysed weight (mg)	10.0
Theoretical % of water	11.88

Table S7. Karl Fischer analysis of **FSTz·4H₂O**

Thermogravimetric Analysis (TGA)

TGA was carried out on a Mettler-Toledo TGA 2 Star System. 3.4132 mg of **FSTz·4H$_2$O** were placed in an alumina pan and heated under a nitrogen flow (50 mL min^{-1}). The heating ramp used was 5 °C min^{-1}, from 30 °C to 400 °C. Figure S23 shows the results of the TGA analysis of **FSTz·4H$_2$O**.

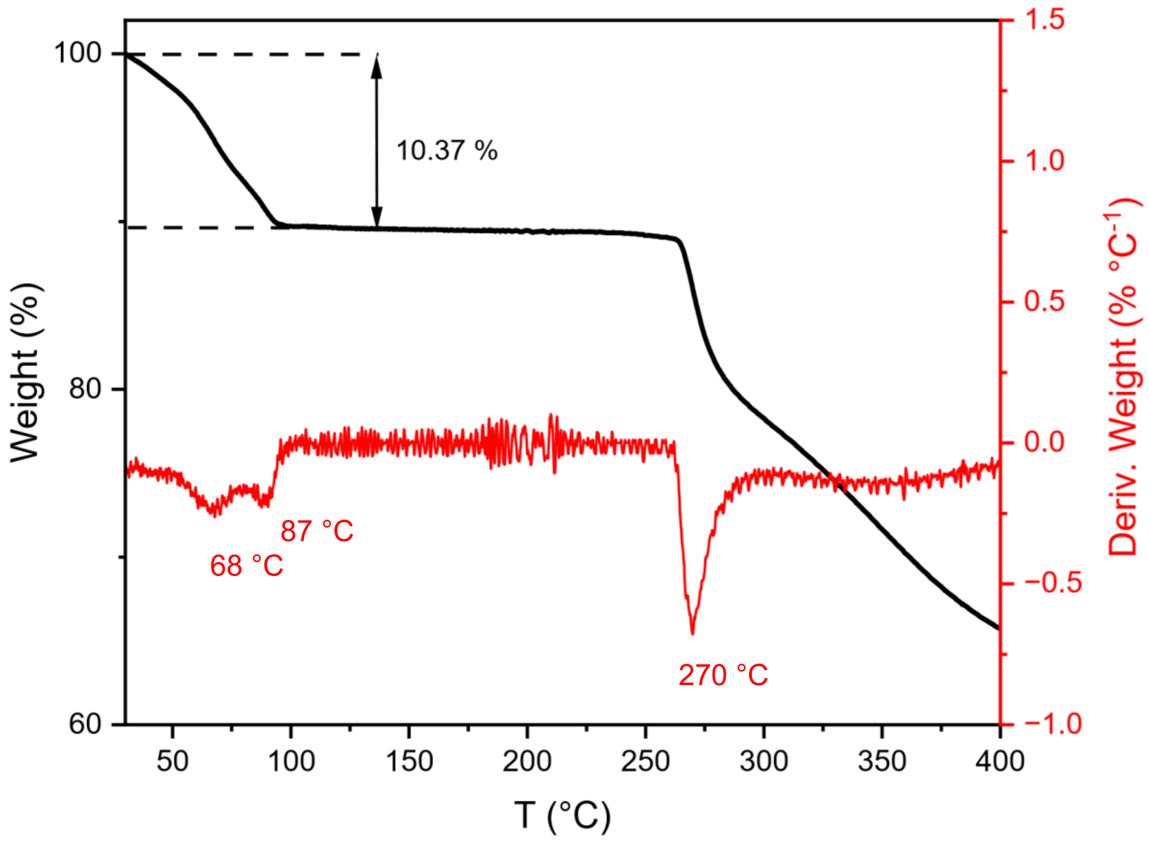

Figure S22. TGA (black line) and DTG (red line) for **FSTz·4H$_2$O**. The continuous mass loss in the range 30-100 °C can be attributed to water molecules desorption. After 250 °C the material starts degrading, T_{dec} = 270 °C (T_{peak}).

Variable-temperature Powder X-ray Diffraction Analysis (VT-PXRD)

The thermal behaviour of **FSTz·4H_2O** was investigated by in situ VT-PXRD using a custom-made sample heater (Officina Elettrotecnica di Tenno, Ponte Arche, Italy) plugged in a Bruker AXS D8 Advance diffractometer. ~20 mg of the powdered bulk sample were deposited in an aluminium sample-holder and heated in air from 303 K until decomposition (T = 723 K) with variable temperature steps (see below). A PXRD pattern was acquired at each step, covering a sensible low-to-medium-angle 2θ range (5.0-30 °, λ = Cu-Kα).

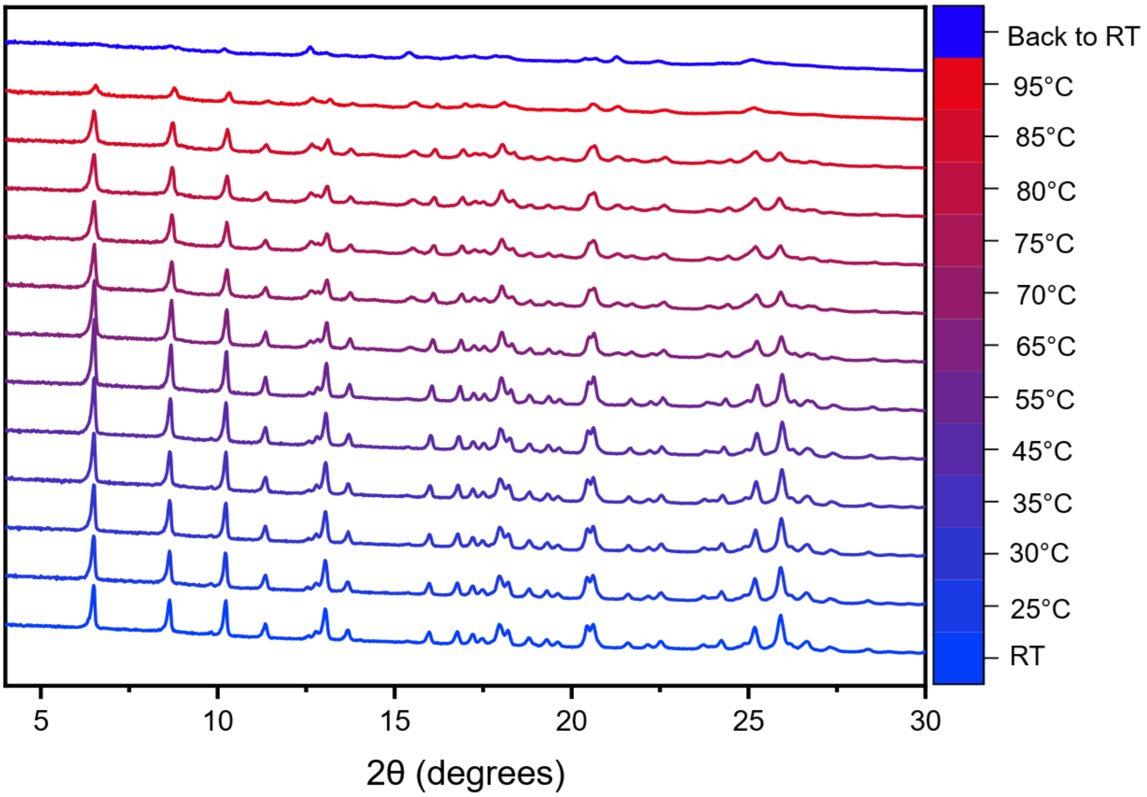

Figure S23. Powder X-ray diffraction patterns measured on **FSTz·4H_2O** sample as a function of ascending temperature in air, with steps of 5 °C, in the temperature range RT (bottom trace, blue) – 95 °C (top trace, red) and back to RT (top trace, blue). This VT-PXRD experiment was performed to detect whether structural changes occur on a **FSTz·4H_2O** sample during the dehydration process, i.e. between RT and 95 °C. No significant changes in the crystalline phase are visible until 85 °C temperature at which the loss of water molecules causes a phase change. The initial tetrahydrate phase is not regained by allowing the sample to return to room temperature (even after prolonged standing in air and humid conditions).

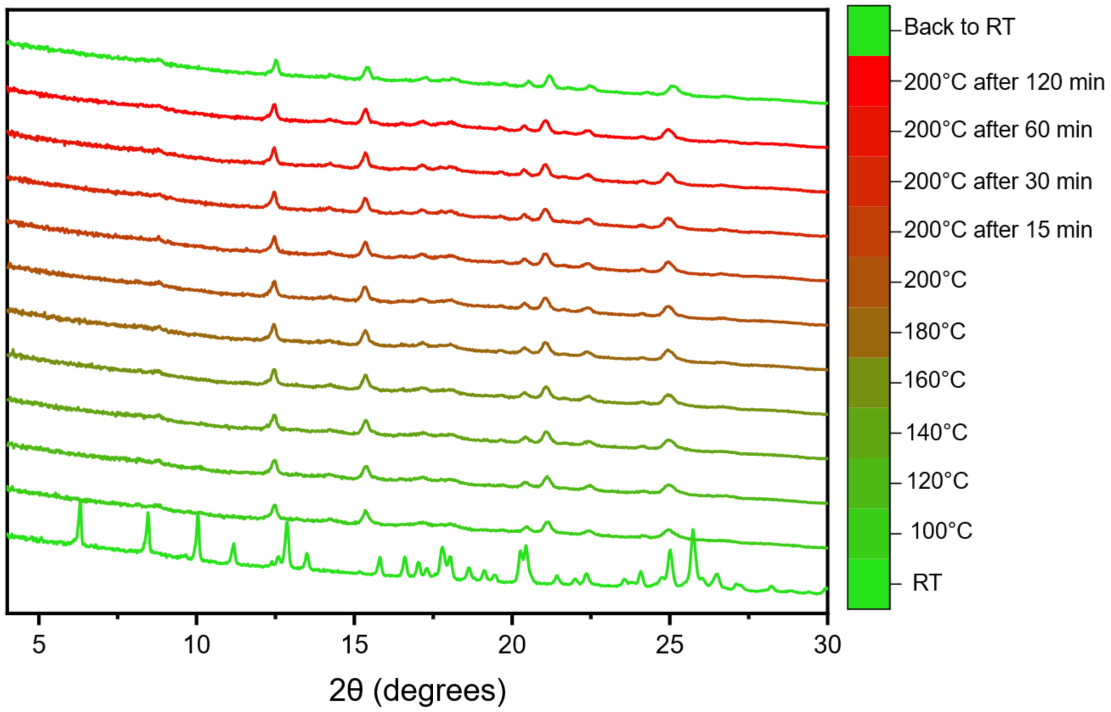

Figure S24. Powder X-ray diffraction patterns measured on **FSTz·4H$_2$O** sample as a function of ascending temperature in air. This experiment was performed to detect the intermediate phases occurring after dehydration. **FSTz·4H$_2$O** was heated to 100 °C with a continuous heating ramp (5 °C/min). The dehydrated sample was then heated to 200 °C with steps of 20 °C and then kept at this temperature for 2 h (top red pattern). After heating, the sample was cooled to room temperature (top green pattern, back to RT).

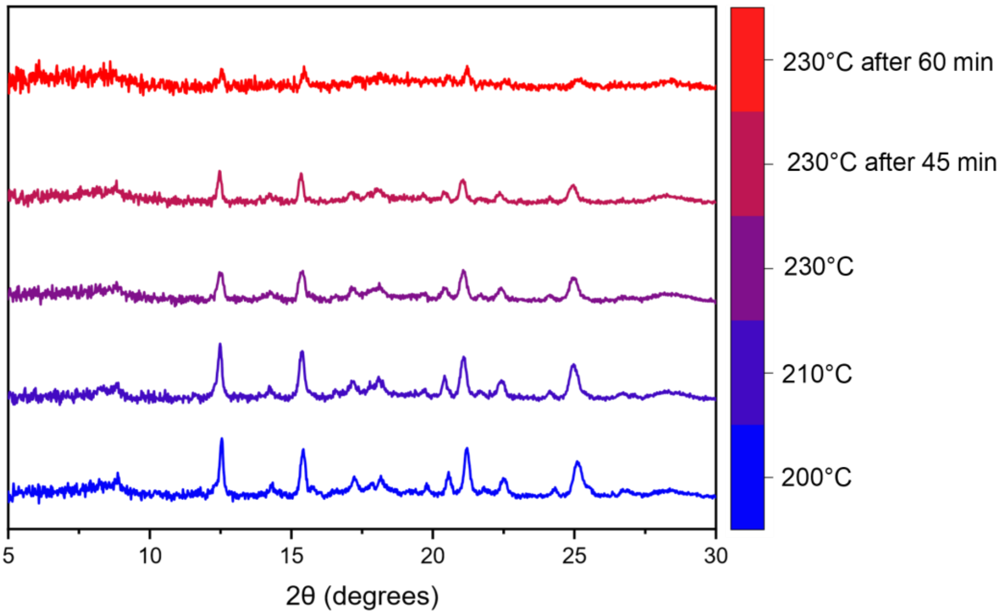

Figure S25. Powder X-ray diffraction patterns measured on the dehydrated FSTz sample as a function of ascending temperature in air. This experiment was performed to understand the decomposition behavior of FSTz. The low-crystalline pattern of amorphous FSTz was heated up to decomposition. No intermediate phases are formed, amorphization occurs with time until complete amorphization.

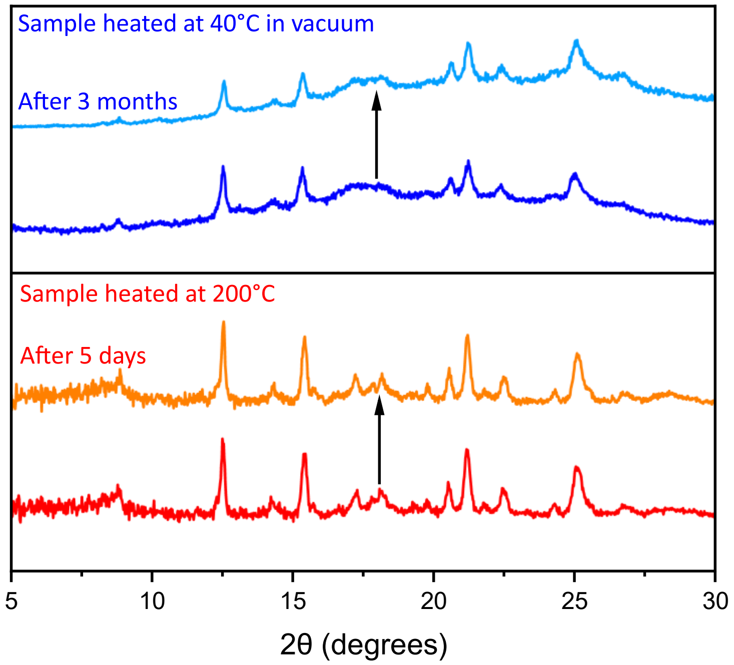

Figure S26. Comparison of PXRD patterns of the dehydrated FSTz samples stored over different period of time. *Bottom plot*: sample after the thermodiffraction experiment (red) and the same sample after storage at room temperature and ambient conditions for 5 days (orange). *Top plot*: sample obtained by heating under vacuum at 40 °C overnight (blue) and the same sample after storage under ambient conditions for 3 months (light blue).

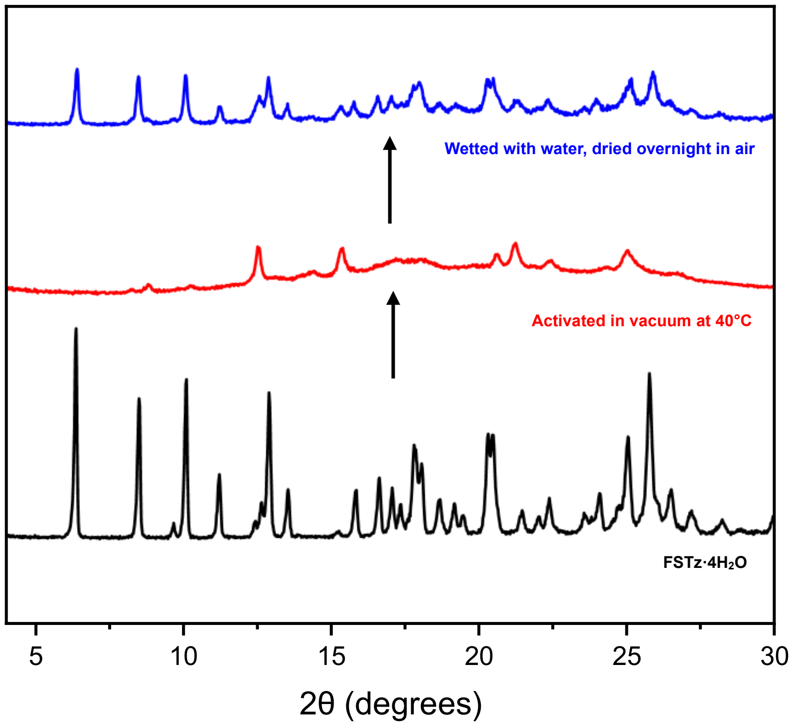

Figure S27. Comparison of powder X-ray diffraction patterns of FSTz·4H_2O (black), the anhydrous phase obtained by heating overnight under vacuum at 40 °C (red), and the same sample after wetting with water in a Petri dish and drying in air overnight (blue). The characteristic crystalline pattern of FSTz·4H_2O is almost fully restored after rehydration. A similar effect is observed when the sample is soaked in water and then dried under vacuum.

Attenuated total Reflection (ATR) Fourier Transform Infrared Spectroscopy (FTIR) analysis.

ATR-FTIR spectra were recorded in the 4000-400 cm^{-1} range at 4 cm^{-1} resolution, using a Bruker Alpha II instrument (Bruker Optics, Rosenheim, Germany) equipped with ATR accessory (monolithic diamond crystal) and DTGS detector. OPUS Software (Release 8.7) was used for the ATR-FTIR spectra processing.

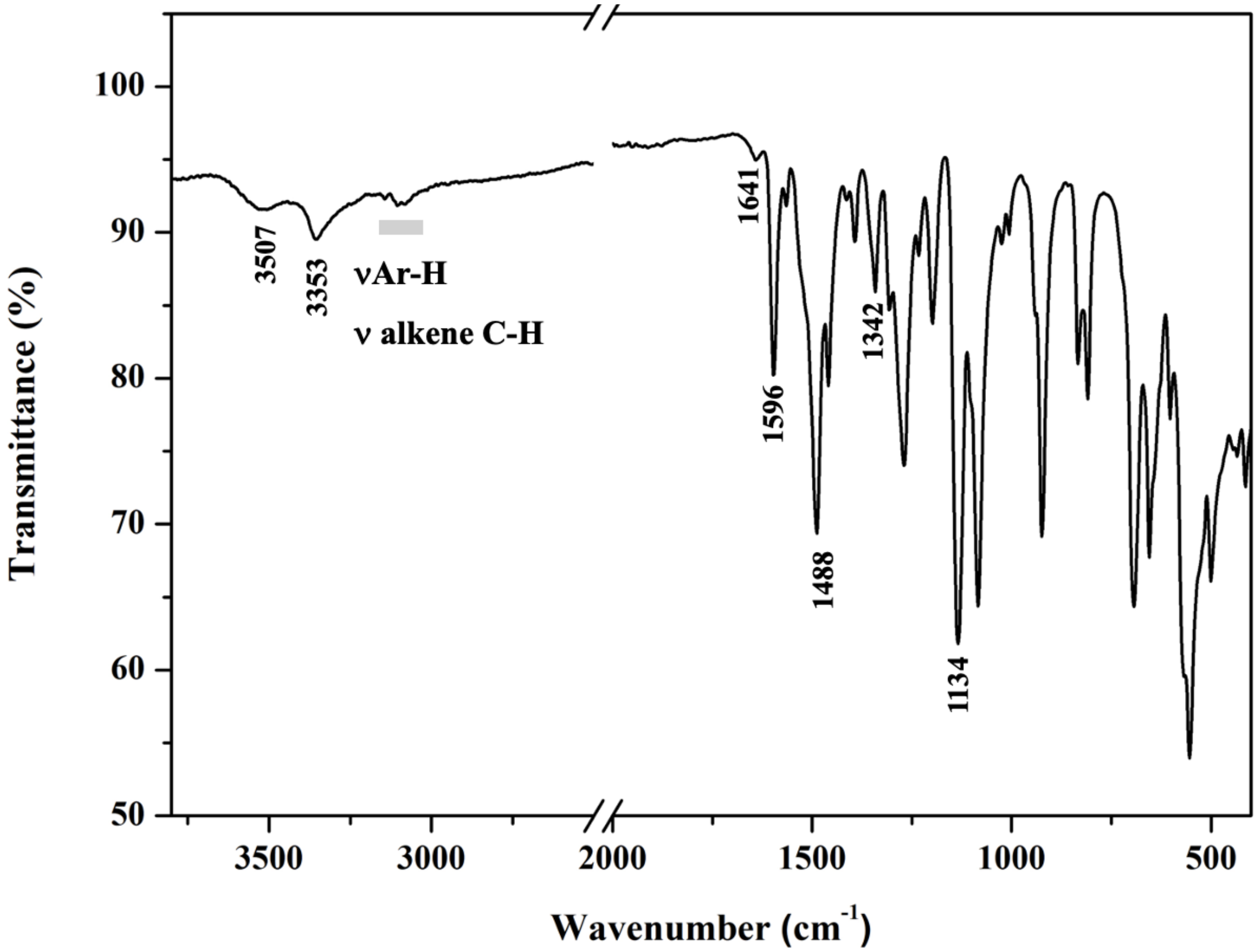

Figure S28. IR spectrum of **FSTz·4H$_2$O**.

In the high frequencies region, FTIR spectrum of **FSTz** is characterized by a broad signal at 3507 cm^{-1} attributed to the stretching mode of water O-H group, a signal at 3353 cm^{-1} attributed to N-H stretching mode of the secondary amine on the benzene ring and by signals around 3140-3100 cm^{-1} due to Ar-H stretching (C-H stretching in benzene rings) and in alkene (C=C-H of thiazole moieties). In the low frequencies' region, ring stretching and C=N stretching are responsible for the signals at 1596 and 1488 cm^{-1}, respectively. Asymmetric and symmetric stretching modes of SO_2 groups can be found at 1342 and 1143 cm^{-1}, respectively.

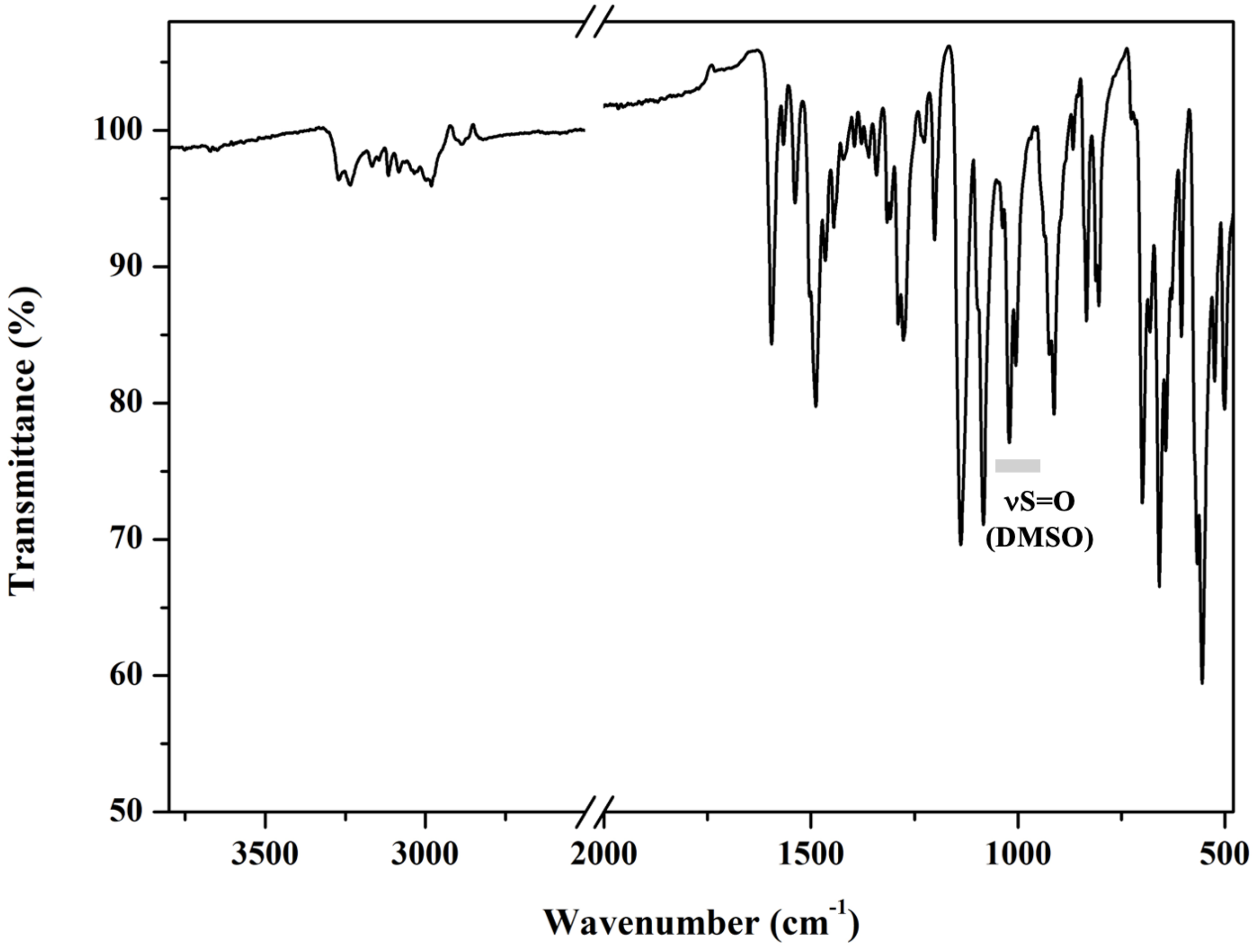

Figure S29. IR spectrum of **FSTz·2DMSO**.

ssNMR

Solid-state magic angle spinning (MAS) NMR spectra were acquired on a Bruker Avance III 500 spectrometer (Fällanden, Switzerland) equipped with a wide bore 11.75 T magnet, operating at frequencies of 500.13 MHz for ^{1}H and 125.77 MHz for ^{13}C. A 4 mm triple-resonance probe, in double resonance mode, with MAS was used in all experiments. The samples were packed on a Zirconia rotor and spun at a MAS rate of 15 kHz for ^{1}H and 13 kHz for ^{13}C. For the ^{13}C cross polarization (CP) MAS experiments, the RF fields of 55 and 28 kHz were used for initial proton excitation and decoupling, respectively. During the CP period the ^{1}H RF field was ramped using 100 increments, whereas the ^{13}C RF fields were maintained at a constant level. During the acquisition, the protons were decoupled from the carbons by using a two-pulse phase-modulated decoupling method. A moderate ramped RF field of 62 kHz was used for spin locking, while the carbon RF field was matched to obtain optimal signal.[12] The relaxation delay between accumulations was 7.5 s and the CP contact time was 2 ms. All chemical shifts are reported using δ scale and are externally referenced to tetramethylsilane at 0 ppm.

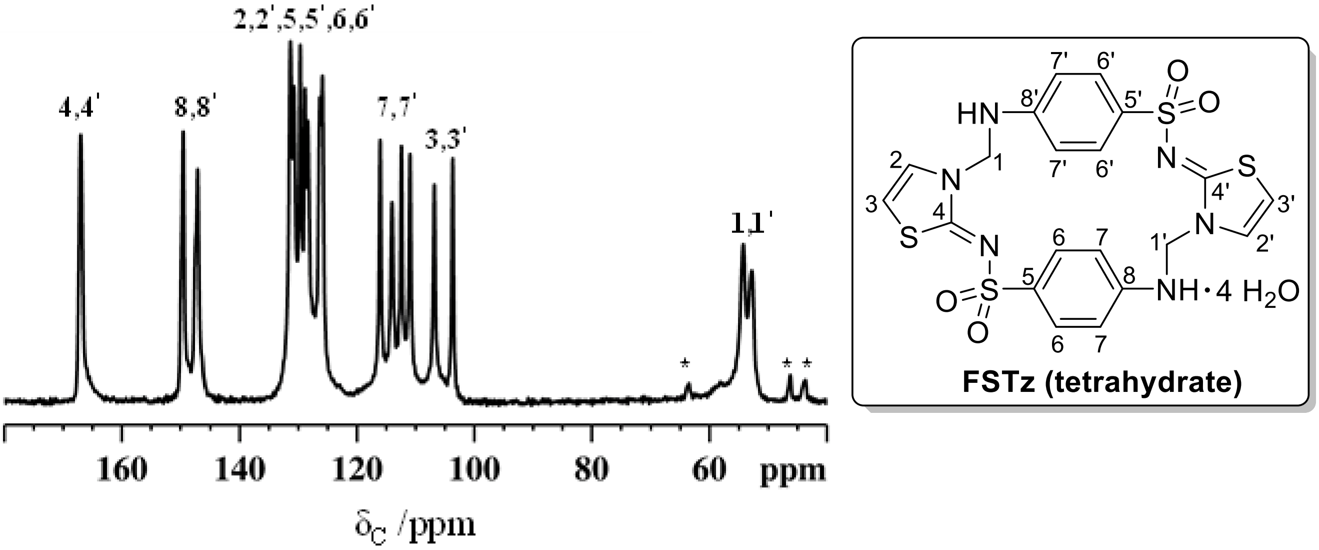

Figure S30. ^{13}C CPMAS NMR spectrum of **FSTz·4H₂O** recorded by using a MAS rate of 13 kHz and a CP contact time of 0.5 ms. Inset shows the molecular structure of **FSTz·4H₂O**. *Spinning sidebands.

**^{13}C** atom number	**δC** / ppm
1, 1'	52.8, 54.3
2, 2'	130.7, 131.3
3, 3'	103.7, 106.8
4, 4'	167.0
5, 5'	129.6, 131.4
6, 6'	125.9, 126.3, 128.4, 128.8
7, 7'	111.0, 112.4, 114.0, 116.0
8, 8'	147.1, 149.5

Table S8. ^{13}C chemical shift data for **FSTz·4H₂O**.

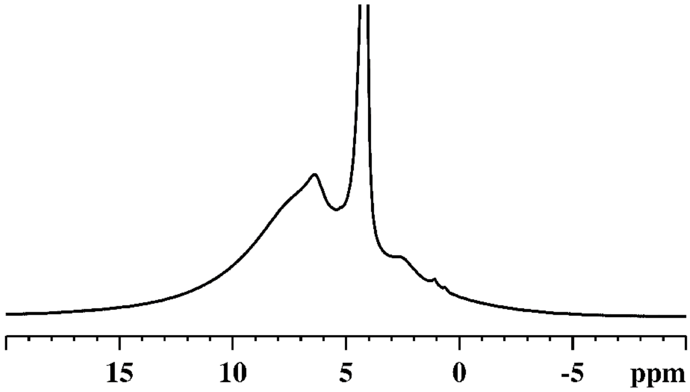

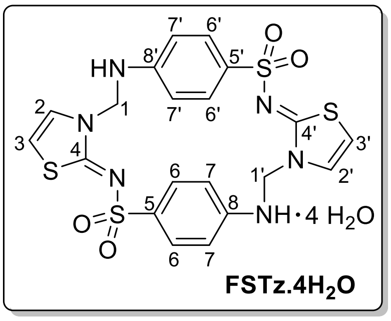

Figure S31. ^{1}H MAS NMR spectrum of **FSTz·4H₂O** recorded by using a MAS rate of 15 kHz. Inset shows the molecular structure of **FSTz·4H₂O**.

**^{1}H** type	Theoretical mol%	Experimental mol% (^{1}H MAS NMR)
N**H**	8	8 ± 5
C**H**$_2$	15	11 ± 5
Ar**H / **Th**H**	46	48 ± 5
**H**$_2$O	31	33 ± 5

Table S9. Relative proportion of the population distribution of ^{1}H sites in **FSTz·4H₂O** derived from the quantitative ^{1}H MAS NMR data analysis.

References

[1] Agilent, CrysAlis PRO, Agilent Technologies Ltd, Yarnton, Oxfordshire, England, **2019**.
[2] G. M. Sheldrick, *Acta Cryst A* **2015**, *71*, 3–8.
[3] L. J. Farrugia, *J Appl Cryst* **2012**, *45*, 849–854.
[4] G. M. Sheldrick, *Acta Cryst C* **2015**, *71*, 3–8.
[5] A. A. Coelho, *J Appl Cryst* **2017**, *50*, 1323–1330.
[6] "TOPAS-Academic," can be found under http://www.topas-academic.net
[7] A. A. Coelho, *J Appl Cryst* **2018**, *51*, 210–218.
[8] A. Le Bail, H. Duroy, J. L. Fourquet, *Materials Research Bulletin* **1988**, *23*, 447–452.
[9] R. W. Cheary, A. Coelho, *Journal of Applied Crystallography* **1992**, *25*, 109–121.
[10] F. H. Allen, I. J. Bruno, *Acta Cryst B* **2010**, *66*, 380–386.
[11] C. F. Macrae, I. Sovago, S. J. Cottrell, P. T. A. Galek, P. McCabe, E. Pidcock, M. Platings, G. P. Shields, J. S. Stevens, M. Towler, P. A. Wood, *J Appl Cryst* **2020**, *53*, 226–235.
[12] G. Paul, S. Steuernagel, H. Koller, *Chem. Commun.* **2007**, 5194–5196.

Author Contributions

Following Contributor Roles Taxonomy (CRediT)

Conceptualization (Ideas; formulation or evolution of overarching research goals and aims)
GBG, VC
Data curation (Management activities to annotate (produce metadata), scrub data and maintain research data (including software code, where it is necessary for interpreting the data itself) for initial use and later re-use)
N/A
Formal analysis (Application of statistical, mathematical, computational, or other formal techniques to analyse or synthesize study data)
CM, TG, FT, CG, MC, IM, GP, SZ, MV, VC
Funding acquisition (Acquisition of the financial support for the project leading to this publication)
GBG, VC
Investigation (Conducting a research and investigation process, specifically performing the experiments, or data/evidence collection)
CM, TG, FT, CG, MC, IM, GP, SZ, MV
Methodology (Development or design of methodology; creation of models)
N/A
Project administration (Management and coordination responsibility for the research activity planning and execution)
GBG, VC
Software (Programming, software development; designing computer programs; implementation of the computer code and supporting algorithms; testing of existing code components)
N/A
Resources (Provision of study materials, reagents, materials, patients, laboratory samples, animals, instrumentation, computing resources, or other analysis tools)
GBG, MC, VC
Supervision (Oversight and leadership responsibility for the research activity planning and execution, including mentorship external to the core team)
GBG, VC
Validation (Verification, whether as a part of the activity or separate, of the overall replication/reproducibility of results/experiments and other research outputs)
N/A
Visualization (Preparation, creation and/or presentation of the published work, specifically visualization/data presentation)
TG, FT, SZ, MV, VC
Writing – original draft (Preparation, creation and/or presentation of the published work, specifically writing the initial draft (including substantive translation).
GBG
Writing – review & editing (Preparation, creation and/or presentation of the published work by those from the original research group, specifically critical review, commentary or revision – including pre- or post-publication stage)
All authors
